# Transcriptome analysis of contrasting resistance to herbivory by *Empoasca fabae* in two shrub willow species and their hybrid progeny

**DOI:** 10.1371/journal.pone.0236586

**Published:** 2020-07-29

**Authors:** Wanyan Wang, Craig H. Carlson, Lawrence B. Smart, John E. Carlson

**Affiliations:** 1 Ecosystem Science and Management, Pennsylvania State University, University Park, Pennsylvania, United States of America; 2 Horticulture Section, School of Integrative Plant Science, Cornell University, Cornell AgriTech, Geneva, New York, United States of America; Austrian Federal Research Centre for Forests BFW, AUSTRIA

## Abstract

Short rotation woody biomass cultivars developed from fast-growing shrub species of willow (*Salix* spp.) have superior properties as perennial energy crops for the Northeast and Midwest US. However, the insect pest potato leafhopper (PLH) *Empoasca fabae* (Harris) can cause serious damage and reduce yield of susceptible genotypes. Currently, the willow cultivars in use display varying levels of susceptibility under PLH infestation. However, genes and markers for resistance to PLH are not yet available for marker-assisted selection in breeding. In this study, transcriptome differences between a resistant genotype 94006 (*S*. *purpurea*) and a susceptible cultivar ‘Jorr’ (*S*. *viminalis*), and their hybrid progeny were determined. Over 600 million RNA-Seq reads were generated and mapped to the *Salix purpurea* reference transcriptome. Gene expression analyses revealed the unique defense mechanism in resistant genotype 94006 that involves PLH-induced secondary cell wall modification. In the susceptible genotypes, genes involved in programed cell death were highly expressed, explaining the necrosis symptoms after PLH feeding. Overall, the discovery of resistance genes and defense mechanisms provides new resources for shrub willow breeding and research in the future.

## Introduction

The increasing worldwide demand for energy, together with the rise in atmospheric greenhouse gases and accumulating evidence of climate change, compels the development of renewable energy sources. Fast-growing shrub willow species (*Salix* spp.) have shown great potential for development of short rotation woody biomass crops for renewable and sustainable bioenergy sources as alternatives to traditional petroleum energy and ‘first generation’ biofuels produced from annual crops [1]. As a feedstock for biofuels and bioproduct, shrub willow has multiple advantages: fast-growing with a short harvest cycle, high biomass yield, high net energy ratio, and relatively low demand for fertilizer and management inputs [2]. Willow can also adapt to harsh field conditions, like marginal agricultural land [3], and it has favorable environmental impacts such as soil remediation and land reclamation [4].

The family Salicaceae is largely comprised of members of the genera *Salix* and *Populus*. Although major genomic rearrangements have occurred since the divergence of these genera, their genomes are largely collinear [5]. There are over 300 species of *Salix* worldwide, which mainly grow in temperate and arctic areas in the northern hemisphere [6]. The genus *Salix* has high levels of genetic diversity due to speciation often involving interspecific hybridization and polyploidy. Diversity in willow genetic resources provides potential for developing improved cultivars with desirable traits like increased yield, lower incidence of rust infection (*Melampsora* spp.), etc. [7].

Potato leafhopper (*Empoasca fabae* Harris) (PLH) is an insect pest in the eastern and midwestern US and parts of eastern Canada [8, 9] and cause tremendous damage to shrub willow. In general, PLH populations originate in the Gulf Coast and the southeastern US and then migrate up to the northeastern states, where their arrival time and development rate are affected by weather conditions and host species availability [10]. The hosts of PLH include over 220 species of plants in 26 families [11], but the primary host is alfalfa, as well as potatoes and legumes [12]. Both adults and nymphs can cause damage to susceptible genotypes of shrub willow via their lacerate-and-flush feeding behavior [13], which consists of continuously rupturing cells (80% of its probing time), secreting watery saliva, and ingesting the plant fluids from phloem tissues [14]. Studies on alfalfa (*Medicago sativa* L.) plants injured by PLH identified a cascade of anatomical changes on stem vascular tissues within a few minutes of laceration [15, 16]. The damage starts with the rupturing, crushing, and later blockage of phloem cells [15], along with increased cell division with atypical planes and development of wound phloem transfer cells, which are similar to callus tissue [16]. The disorganization of vascular bundles causes symptoms on plants called “hopperburn”, which is a sequence of abnormal states: tip dehydration and wilting, leaf chlorosis, early leaf drop, as well as internodal growth restriction and consequential stunting of growth [17]. The most direct and serious damage of PLH to willow biomass yield is the stunted plant growth [18] and weakened plant defenses, especially under other stress conditions such as drought. Chemical insecticides are used to control PLH on other host crops [19], whereas no agrochemical management is currently deployed on shrub willow due to the extra cost and concern for environmental sustainability. Thus, development of resistant cultivars of shrub willow is required for successful commercial deployment. Shrub willow genotypes can vary greatly in susceptibility to PLH. Some are particularly sensitive, suffering significant damage and loss of yield upon PLH feeding. *Salix viminalis*, originating from Europe, harbors desirable physiological traits for high biomass production, which makes it popular among willow breeders and growers [20]. However, this shrub willow species and its derived cultivars show particular susceptibility to the PLH [21, 22], while hybrid crosses of *S*. *viminalis* with *S*. *miyabeana* and *S*. *purpurea* display varying degrees of resistance [23, 24]. It can be challenging to accurately assess resistance to PLH in the field due to variations in pressure across years and because PLH is highly mobile and can make choices toward feeding on its preferred host genotype. In this study, we used potted plants grown in cages in the greenhouse in a no-choice feeding study, the results of which we can compare with field trial surveys to more fully understand the durability of resistance.

Advances in high-throughput sequencing technology greatly facilitate the exploration of the gene expression responses of shrub willow under PLH infestation at the whole transcriptome level. This study compared the transcriptomic differences between two willow species, *S*. *purpurea* (resistant) and *S*. *viminalis* (susceptible) and selected full-sib F_1_ progeny that display varying levels of resistance to PLH at different time points (0, 12, 24 and 96 h after infection) using RNA-Seq. This research provides deeper insights into the defense mechanisms and potential key pathways and genes that determine genotype-specific resistance of shrub willow to PLH feeding, and will help guide the development of improved cultivars for the biofuel industry in the northeast US.

## Materials and methods

### Plant and pest materials

An F_1_ hybrid family, 11X-407, was generated by crossing female *S*. *purpurea* 94006 with *S*. *viminalis* ‘Jorr’, a cultivar bred in Sweden. Progeny in the F_1_ family and their parents were planted in a field trial in Geneva, NY, USA, in 2014 in which each genotype was planted in a three-plant plot in each of four randomized complete blocks. The spacing was a single-row design with 1.83 m between rows and 0.46 m spacing between plants within a row. PLH damage was surveyed that growing season (see details below) and after coppice during the 2015 growing season. Plant height was measured after each growing season as well. Based on the field surveys results, 18 hybrid progeny genotypes displaying a wide range of PLH susceptibility were selected for the subsequent no-choice feeding trial in a greenhouse, together with parents 94006 and ‘Jorr’. Dormant cuttings (20 cm) were collected in March 2015 from one-year-old stems of plants growing in nursery beds and were planted in potting mix on June 9, 2015 with one cutting in each of four replicate 15 cm (~3 L) pots per genotype (80 pots in total). The four replicate pots of each genotype were grown inside a fine mesh cage (60 × 60 × 120 cm, Bugdorm 6620) in a greenhouse with a daytime (14 h supplemental lighting) temperature of 26–28°C and nighttime temperature of 20–22°C. A few cuttings that did not break bud were replaced with new cuttings within 7–10 d. Only three plants of the ‘Jorr’ parental genotype could be obtained in this manner for the experiment.

PLH adults were collected from nursery beds of susceptible cultivars ‘Klara’ and ‘Stina’ using a gas-powered vacuum (modified leaf blower) fitted with a mesh bag. The insects were then sedated with CO_2_ and ca. 20 to 30 individuals were put into each of 20 vials with moist cotton and a fresh leaf and kept in a growth chamber overnight prior to starting the feeding trial.

### Greenhouse no-choice feeding experiments

Approximately 20 to 30 PLH adults in a single vial were released into each cage starting at 9 am on Day 1. Additional PLH were introduced the next day to substitute for any that had died. Leaf samples were collected according to a time course: time 0 (before treatment), time 6 h, time 24 h, and time 96 h after PLH introduction. This time course was chosen because the leaf curling symptoms were observed within only a few hours after PLH introduction. For tissue sampling, a single young, expanded leaf was collected near the top of a dominant shoot of each plant, folded and pushed into a 2 mL grinding tube, and quick frozen in liquid nitrogen, then stored in a -80°C freezer. In the later time points, those leaves may have been showing symptoms of PLH damage and were not avoided in sampling. Plants were watered as needed through the mesh portals of the cages.

### Phenotype measurements

In the greenhouse experiment, observable plant characteristics or traits were measured and recorded in response to PLH exposure over time. By using the previous observation of severely damaged tissue as the 100% damage standard, damage severity (%) was visually scored to reflect tip necrosis, leaf curl and leaf yellowing as a survey of the shoot tip region of each plant for all plants at 4 d of PLH exposure (4 d) and again after 11 d. Stem lengths of each plant were measured at the time of PLH introduction and again after 11 d, to calculate the percentage change of stem length as an indicator of PLH impact on stem elongation. As many as 4 to 5 living PLH adults were found remaining in every cage at the end of the experiment. Nymphs were observed on plants of three genotypes 11X-407-085, 11X-407-070, 11X-407-102, indicating that the PLH were not only able to feed on these genotypes, but also to complete their life cycle through oviposition and production of new nymphs. However, these three genotypes were among the least damaged by PLH in many previous pest surveys.

During 2014 and 2015, all genotypes (parents and progeny) were surveyed for various phenotypic traits in field trials. Three characteristic PLH infection symptoms–shoot tip damage, leaf necrosis and chlorosis, and leaf curl–were surveyed according to an established rubric three times (August 2014, September 2014 and September 2015). Stem heights were also measured at three time points (December 2014, June 2015 and August 2015). Mean phenotype measurements were calculated for each genotype and the means used as phenotypic traits for each time point in Weighted Gene Correlation Network Analysis (WGCNA).

### RNA extraction and sequencing

Total RNA was extracted from each leaf sample using a Spectrum^™^ Plant Total RNA Kit (Sigma-Aldrich, St. Louis, MO) and checked for quality using an Agilent 2100 Bioanalyzer (Agilent Technologies, Santa Clara, CA 95051, USA) at the Penn State Genomics Core Facility University Park, PA. RNA-Seq bar-coded libraries were prepared on a robotic platform using the Illumina Trueseq Library Kit 2 for each of the 96 total RNA samples of highest quality (RNA integrity number (RIN) > 6) and sequencing was conducted on an Illumina HiSeq 2500 sequencer at the Singapore Centre for Environmental Life Sciences Engineering at the Nanyang Technological University. Two nucleotide paired end (2×101bp) sequencing runs were conducted, yielding a total of 613 M reads for the 96 libraries. Data files in fastq format were provided for subsequent RNA-Seq data analysis. Sequence data are archived at National Center for Biotechnology Information BioProject ID PRJNA601117.

### Calculation and quantification of gene expression abundance

The raw sequencing reads were trimmed and filtered using Trimmomatic v0.36 [25], and mapped to the *S*. *purpurea* reference genome predicted transcript sequences (*Salix purpurea* v1.0, DOE-JGI, Phytozome 12.0 https://phytozome.jgi.doe.gov/pz/portal.html#!info?alias=Org_Spurpurea) using Bowtie2 version 2.2.8 [26], with parameters set to—*local* and—*sensitive* mode. The generated bam alignment files were processed with Samtools [27] ‘idx’ function to count the number of mapped reads per transcript. Results were stored in matrix format for further analyses.

### Differential gene expression analysis

The R package DESeq2 version 1.10.1 [28] was used to determine statistically significant differential expression using a model based on the negative binomial distribution. Principal component analyses (PCA) were also calculated with the DESeq2 package. For the false discovery rate controlling, Benjamini and Hochberg’s approach [29] was implemented. Thresholds combining false discovery rate (FDR) < 0.001 and absolute value of log_2_ ratio ≥ 1 were used to define significant differentially expressed genes (DEGs) in this study. Venn diagrams of differentially expressed genes were generated using web application BioVenn [30].

### Weighted gene correlation network analysis (WGCNA)

The R package WGCNA version 1.36 [31] was used to identify modules of genes shared highly-correlated expression patterns. The low-expressed genes with read count < 5 for 80% of all the libraries were filtered before subjected to WGCNA clustering. Raw counts were normalized using the *varianceStabilizingTransformation* function in DESeq2 [27]. A soft threshold value, power of 9, was used to transform the adjacency matrix to meet the scale-free topology criteria for optimal clustering. The outlier libraries were identified using an average linkage hierarchical cluster tree based on Euclidean distance. Modules of genes with correlated expression were obtained using a stringency threshold of 0.75. To understand the physiologic significance of each module, 25 module eigengene expression profiles were correlated with physiological traits from both field and greenhouse trials such as pest damage and growth rate, generating a full module-trait correlation table. For inheritance analysis, only genes with a sum normalized CPM > 1 for ≥ 50% of the samples were considered prior to network construction.

### Gene ontology (GO) and Kyoto Encyclopedia of Genes and Genomes (KEGG) pathway analysis

*Salix* gene IDs were first transferred to *A*. *thaliana* based on the gene annotation file from the *Salix purpurea* reference genome v1.0, DOE-JGI, assigned with IDs from TAIR (The Arabidopsis Information Resource) database. DEGs with corresponding TAIR IDs were subjected to GO term singular enrichment against *Arabidopsis* background from *AgriGo* database v1.0 [32]. Fisher’s exact test was used for the enrichment analysis and the Bonferroni method was applied to evaluate the FDR with the significance level set to 0.05. DEGs were also subjected to KEGG enrichment analysis (http://www.genome.jp/kegg) [33] to identify the statistical enriched KEGG pathways with FDR < 0.05. Software MapMan (http://mapMan.gabipd.org) [34] was used to display log fold change of expressions between both parent genotypes on the cell function and metabolism overview maps.

### Inheritance of gene expression

To determine the mode of inheritance for genes, the number of RNA-Seq reads mapped to individual genes was counted for each of the female (P1) and male (P2) parents and F_1_ progeny (H). DEGs (FDR = 0.01) were determined using an exact test implemented in edgeR [35] for negative-binomially distributed counts. Only genes with a sum normalized counts-per-million (CPM) > 1 for at least 50% of the samples were considered in analyses. A custom R script was used to sort genes into the following six inheritance categories: (1) P1-dominant: H≈P1 and H≠P2, (2) P2-dominant: H≈P2 and H≠P1, (3) additive: P1<H<P2 or P2<H<P1, (4) overdominant: H>P1 and H>P2, (5) underdominant: H<P1 and H<P2, and (6) conserved: P1≈H≈P2. Manhattan plots were generated using an R package ‘qqman’, to graphically show the significant associations between genes and phenotypic traits on a genomic scale. The plot is produced by scattering the scaled *p*-values in the format of: |R^2^| × −log_10_ (*p*-value) on the vertical axis and the physical position of each gene across 19 chromosomes on the horizontal axis. The value of -log_10_ (*p*-value = 0.05/*n* genes) was chosen as threshold to reach the genome-wide significance.

### Validation of gene expression with real-time quantitative PCR in parent genotypes

To validate accuracy of the RNA-Seq expression profiles, six genes encoding transcription factors (TFs), ligandins and heat shock protein in both willow genotypes (94006 and ‘Jorr’), across four time points, were selected for quantitative real-time PCR analysis. cDNA was synthesized from the total amount of RNA using the iScript cDNA synthesis kit (Bio-Rad, Nazareth), and the primers for qPCR were designed using the IDT PrimerQuest Primer Design Tool and synthesized by IDT. The qPCR was performed using the iTaq^™^ Universal SYBR^®^ Green Supermix (Bio-Rad Laboratories, CA, USA) on the BioRad CFX96^™^ Real-Time PCR system (Bio-Rad Laboratories, CA, USA). Relative gene expression was calculated using the comparative Ct method (2^−ΔΔCt^) and normalized to that of the endogenous reference—the membrane-anchored ubiquitin-fold protein—MUB (SapurV1A.2454s0040). The qPCR expression patterns of the six genes in both willow genotypes (94006 and ‘Jorr’), across four time points, were compared to RNA-Seq normalized expression profile.

## Results

### Phenotypic responses of shrub willow to PLH attack in greenhouse and field trials

**Fig 1:**
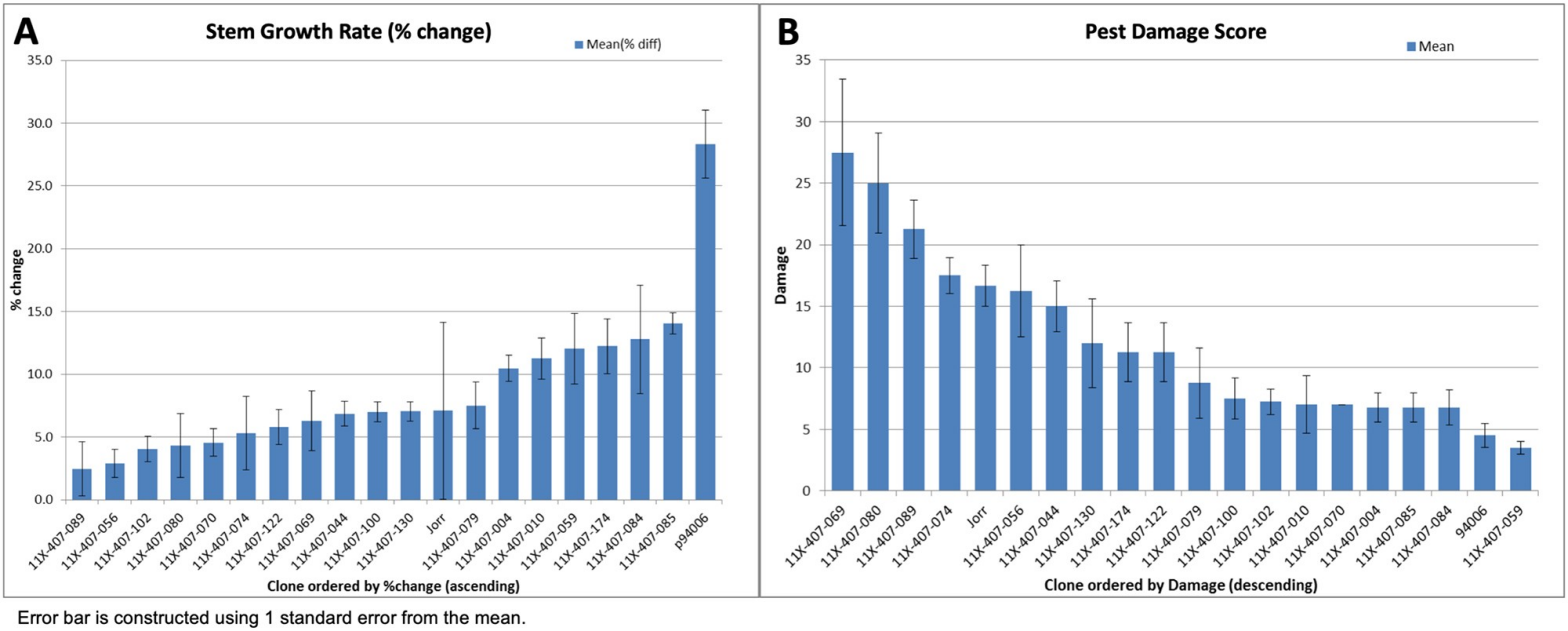
Greenhouse phenotypic measurements of stem elongation rate and damage visual scoring. A) Mean change in total stem length (%) per plant over the 11 d of PLH exposure in the no-choice greenhouse feeding trial. Length of all stems per plant was measured with a meter stick at time 0 and time day 11 when the experiment ended. The percent change in total stem length per plant was calculated to reflect the effect of PLH on stem elongation over the 11 days. B) Damage severity (%) was scored for each plant after 11 days of PLH exposure, and averaged for each genotype.

In a greenhouse no-choice feeding trial, symptoms of PLH feeding could be observed on some plants within hours. Leaf curling started before the first tissue collection point at 6 h. Exposure to PLH feeding also resulted in differences in stem elongation over the 11-day period (Fig 1A). The resistant parent, *S*. *purpurea* 94006, added a mean of 28.3% in total stem length per plant at the end of the experiment (day 11), while the susceptible parent, *S*. *viminalis* cv. ‘Jorr’, increased only a mean of 7.1% in total stem length. The percent change among progeny varied from 14.1% for genotype 11X-407-085 to only 2.5% for genotype 11X-407-089, with an overall mean of 7.6% for all hybrid progeny. There were also differences in pest damage severity (shoot tip and young leaf necrosis) by genotype (Fig 1B), with a mean scaled percentage of 4.5% on 94006 and 16.7% on ‘Jorr’. The damage severity among the progeny varied from 3.5% for genotype 11X-407-059 to 27.5% for genotype 11X-407-069, with a mean among all progeny of 12.1%. There was a significant negative relationship (R^2^ = 0.16, *p*-value = 0.0004) between the stem elongation rate and the severity of damage.

The variation of PLH attack symptoms observed among genotypes in the greenhouse no-choice feeding trial was generally consistent with performance in the field trials where PLH adults could freely make host choices. Survey data from mid-season 2014 and 2015 collected from field trials of 100 F_1_ progeny were used to select 18 progeny individuals that represented the full range of susceptibility, scored as shoot tip necrosis, leaf curling, and stem height. There was a significant positive correlation (R^2^ = 0.24, *p*-value < 0.0001) between height measurements in the field in 2015 and stem growth rate in the greenhouse no-choice feeding trial. There is also a significant negative correlation (R^2^ = 0.18, *p*-value = 0.0001) between shoot damage severity scored in the field in 2014 and stem growth rate in the greenhouse. Based on both damage survey and growth measurement results, seven progeny were selected that covered a wide range of susceptibilities for transcriptome sequencing, along with their parents, 94006 and ‘Jorr’, as controls.

### RNA sequencing and quality assessment

Total RNA was extracted from 96 shrub willow leaf tissue samples, including 2 or 3 replicates of each treatment condition (4 time points × 9 genotypes). RNA sequencing of the 96 mRNA samples on the Illumina HiSeq2500 platform yielded a total of 612,869,652 paired-end reads with length of 101 bp. Library sizes ranged from 4,266,920 to 9,159,176 reads, with a mean of 6,384,059. After trimming and filtering, reads were mapped to the *S*. *purpurea* primary transcript sequences (*Salix purpurea* v1.0, Phytozome 12.0 DOE-JGI) using Bowtie2 version 2.2.4 [26]. Mapping rates ranged from 68.99% to 81.67%.

### The transcriptomes of parents and F1 hybrids were differentiated by genetic background and timing after infestation

**Fig 2:**
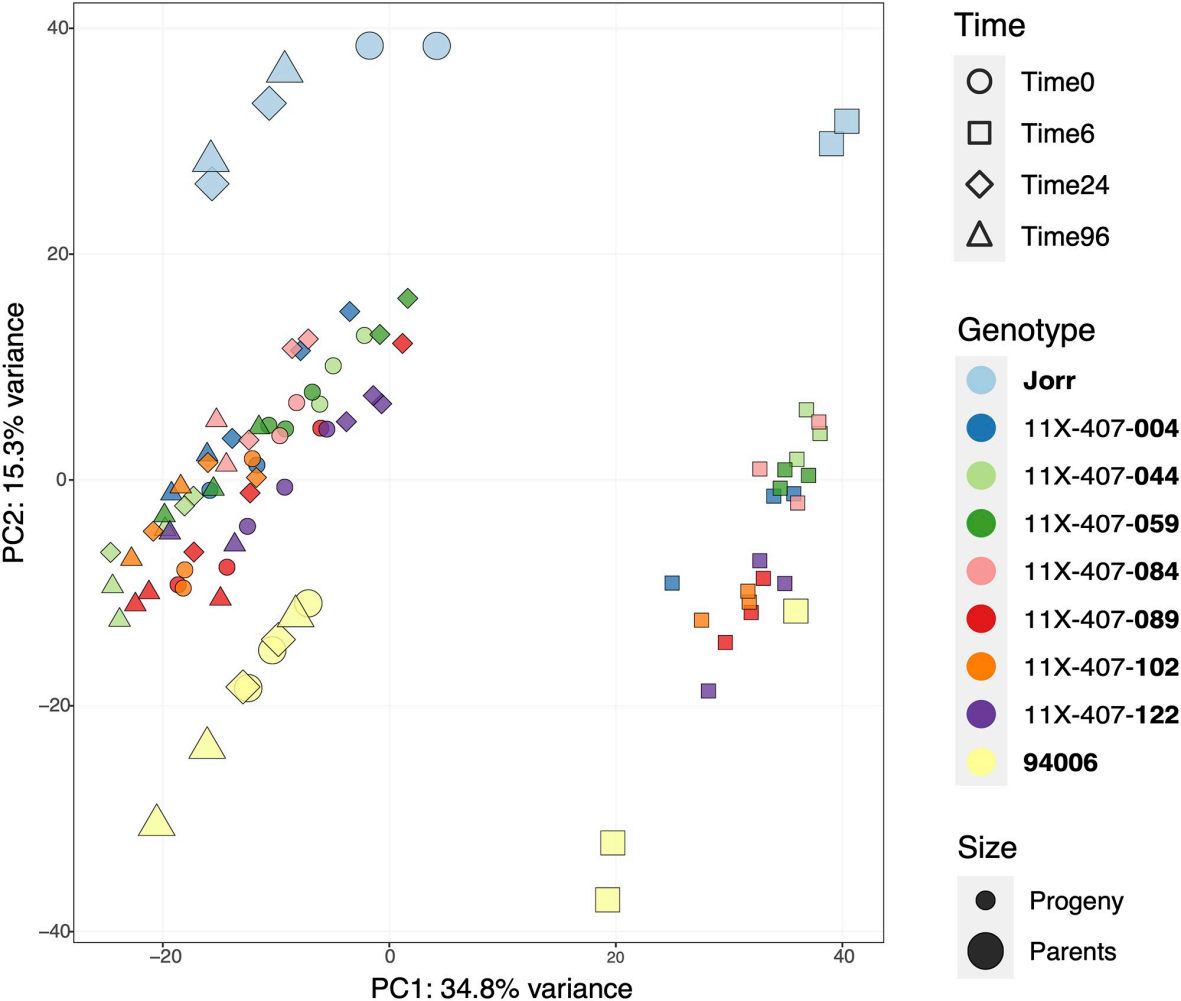
PCA plot of all samples of the two parent species and seven progeny genotypes at four time points. Parental species and progeny genotypes were depicted using different colors. Different time points were represented as different shapes (Circle: Time 0; Square: Time 6h; Diamond: Time 24h; Triangle: Time 96h). The two parents were marked prominently with larger dots to make it easier to distinguish parents from the parents from the F1 hybrids. The horizontal PC1 dimension, which accounts for 34.8% of the variance among all samples, separated samples among the Time 6h and other time points. The vertical PC2 dimension, which accounts for 15.3% variance of all samples, grouped the parental species separately and the seven progeny genotypes clustered together intermediate to both the parents.

Principal component analysis (PCA) was performed with all samples, including both parental species and the F1 hybrids (Fig 2). The PCA plot displayed the overview of variance among samples from different genotypes and time points. The first principle component, explaining for 34.8% of total variance, separated samples collected at 6 hours after PLH infestation from samples from other time points. This pattern indicated that the most intense defensive response occurred within 6 h, which is in concordance with the phenotypic observation that leaf curling appeared before the first sampling time at 6h after exposure to PLH. The second principal component accounted for 15.3% of the total variance and separated all samples into three groups–parental species were at two ends across this dimension and seven F1 genotypes grouped all together, intermediate to the parents.

### Identification of differentially expressed genes between parents from RNA-seq data

**Fig 3:**
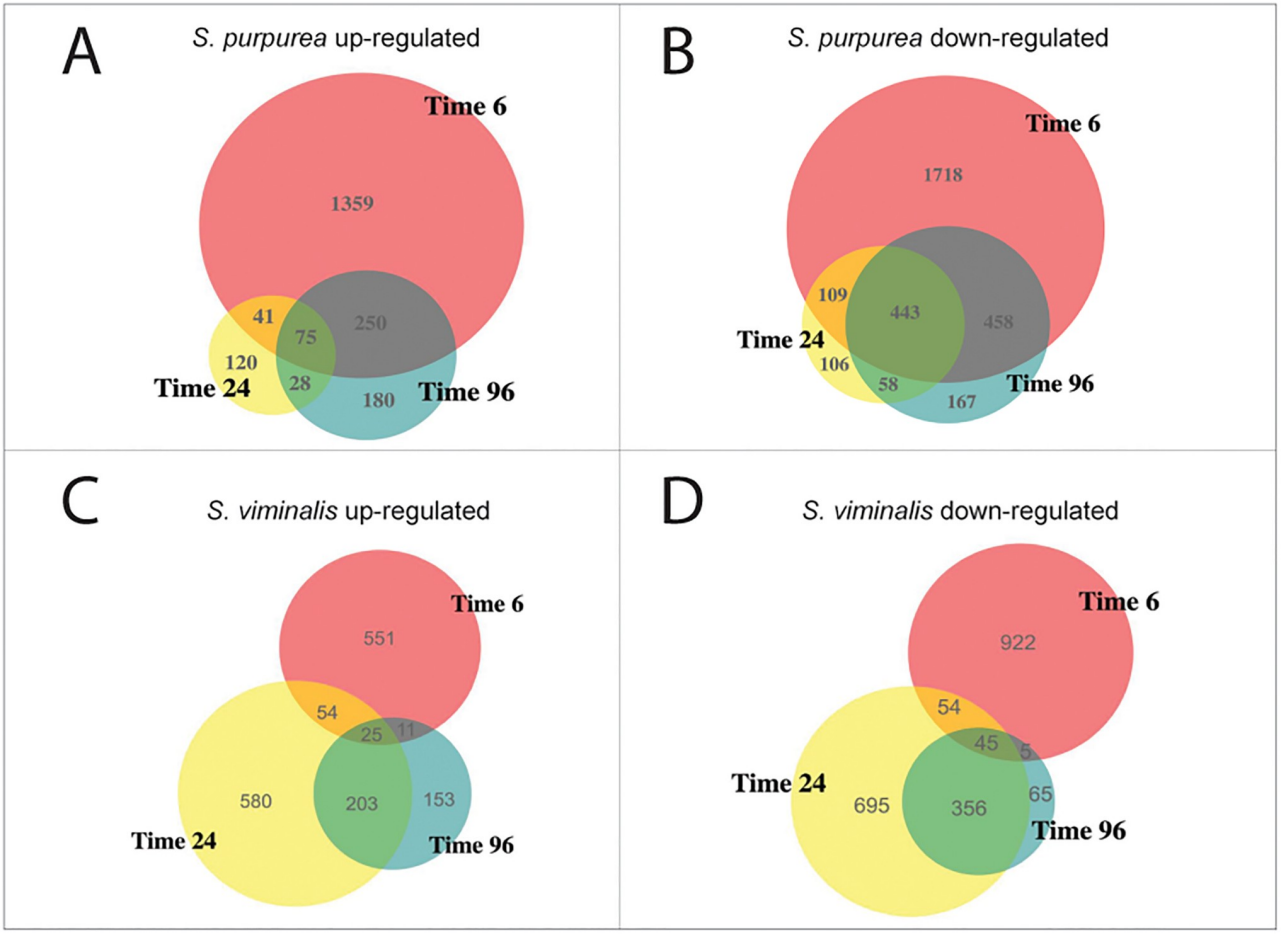
Venn diagrams of up- and down-regulated DEGs via pairwise comparison over uninjured samples. Panel A: *S*. *purpurea* 94006 up-regulated DEGs; Panel B: *S*. *purpurea* 94006 down-regulated DEGs; Panels C: *S*. *viminalis* ‘Jorr’ up-regulated DEGs; Panels D: *S*. *viminalis* ‘Jorr’ down-regulated DEGs. Color represent different pair-wise comparison against reference Time 0 (Red: Time 6h vs Time 0; Yellow: Time 24h vs Time 0; Green: Time 96h vs Time 0).

The identification of differentially expressed genes (DEGs) was implemented with R package DESeq2 [27] via pairwise comparisons of the transcriptomes at different time points relative to time point 0, as the non-treatment control. DEG discovery was based on criteria of false discovery rate (FDR) < 0.05 and expression fold-change > 2. Three sets of DEG (time 6 h vs time 0; time 24 h vs time 0; time 96 h vs time 0) were called separately for both parent genotypes and each set was further split into up- and down-regulated groups. Combining the DEG results for both parents, a total of 6,983 non-redundant DEG (genes belonging to multiple DEG lists were only counted once) were identified from the six pairwise comparisons. Venn diagrams (Fig 3) show relative amounts and overlaps among sets of DEG identified at different time points, with up- and down-regulated genes shown separately for the two parents. As shown in Fig 3, the largest change in gene expression occurred by the 6 h time point for both parents. However, there were more DEG at hour 6 in 94006 (4493) than in ‘Jorr’ (1670), both in terms of total number of DEG and relative to the 24 h time points for both up- and down-regulated genes. As illustrated in the Venn diagram, the greatest differential gene expression changes for genotype 94006 happened at time 6 h, whereas gene expression changes in ‘Jorr’ increase less and were at about the same levels at time 24 h and 6 h, which suggests a more sensitive response, and more rapid initiation of the defensive processes in resistant 94006.

### Weighted Gene Correlation Network Analysis (WGCNA) identified three clusters of genes associated with specific resistance mechanisms

**Fig 4:**
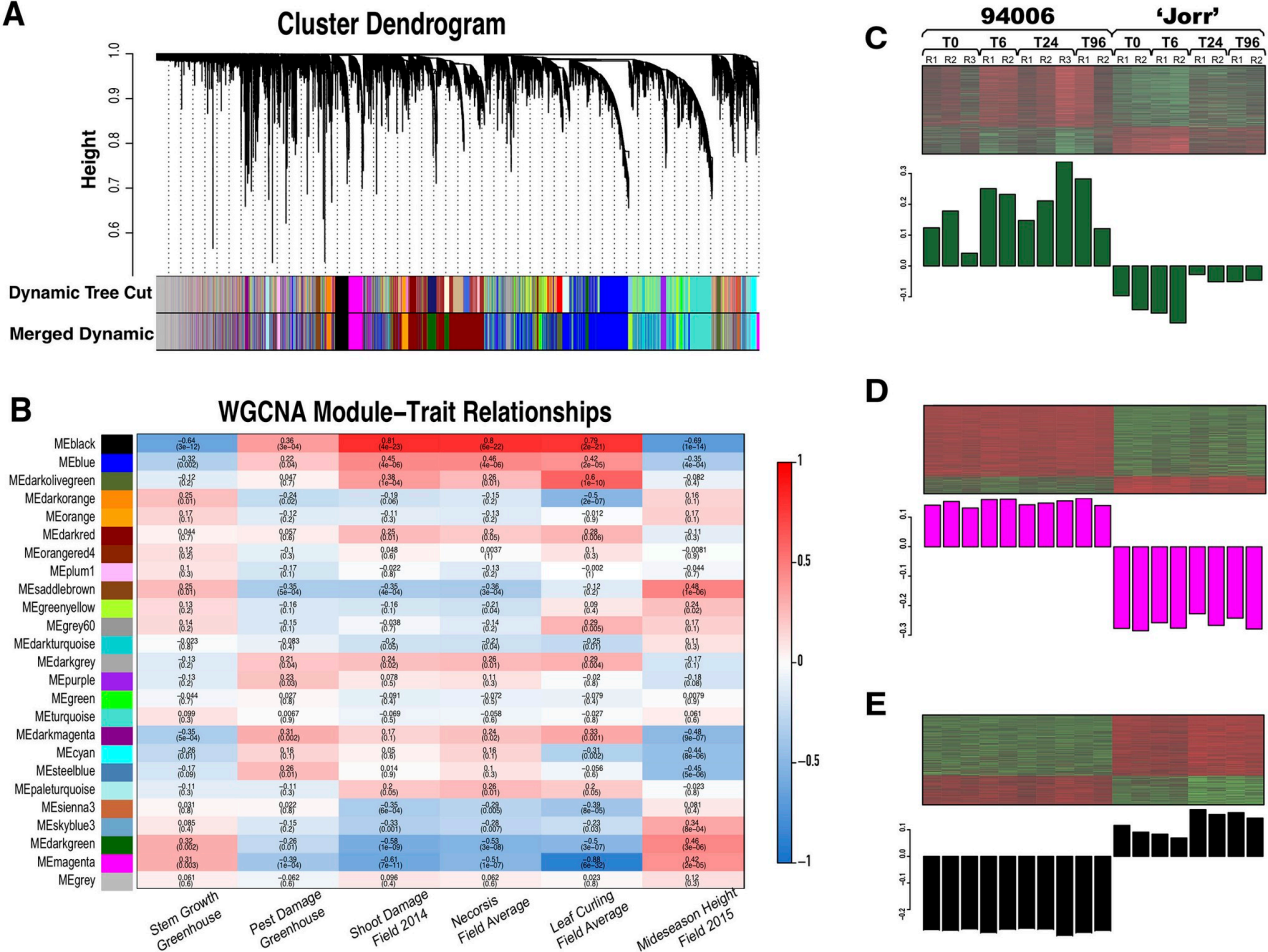
Co-expression analyses of all genes across all samples. A) Hierarchical cluster tree showing 25 modules of co-expressed genes. Each gene is represented by a leaf in the tree. The WGCNA cluster dendrogram on all 96 samples grouped the genes into 25 distinct modules (Row—Merged Dynamic); B) WGCNA Module-trait relationships table. On the left side, each row represents a co-expressed gene cluster, and each column represents data for a phenotype: from left to right–greenhouse stem growth rate; greenhouse pest damage; field shoot damage; field necrosis; field leaf curling; field plant heights. The field damage data (shoot damage, leaf necrosis and leaf curl) were averaged for three different time points for this table. Light color cells within this row indicate low correlation with phenotypes. The right panel shows the heatmaps of gene expression and the eigengene expression profiles across each library of parent genotypes *S*. *purpurea* 94006 and *S*. *viminalis* ‘Jorr’ for C) Module *darkgreen*, D) Module *magenta* and E) Module *black*. The eigengene represents the standardized relative expression levels for each library. Genotype, sampling time point (hat) and biological replicate numbers are indicated above each column in the eigengenes profiles. Columns are vertically aligned for all three modules.

In general, co-expressed genes are likely to be functionally associated and/or dependent, because they belong to common pathways or expression networks. In co-expression analyses using the weighted gene correlation network analysis (WGCNA) approach, genes with tightly correlated patterns of expression are clustered into a co-expressed “module” and arbitrarily assigned a color-based label, as described in Langfelder and Horvath’s paper [31]. Based on the whole dataset, which includes all nine genotypes (2 parents and 7 hybrid progeny) at 4 time points, WGCNA detected 25 gene clusters (stringency threshold = 0.75) (Fig 4A). To interpret the biological function of each gene cluster, eigengene expression values were calculated to summarize the overall expression profile for each module and paired with each set of phenotypic data to analyze the correlation. Included in this study were the phenotypic measurements taken from both the greenhouse and field trials: visual scores for PLH shoot damage, necrosis, and leaf curl at different time points, and stem elongation rates calculated from height data, which were averaged to represent pest susceptibility (see methods). The cluster-trait correlations are depicted in a heat map table in Fig 4B. In this table, three clusters (*darkgreen*, *magenta*, *black*) displayed consistently strong correlations (either positive or negative) with all phenotypic traits, indicating their potential functional linkage with PLH-resistance and growth traits. The genes that cannot be assigned to any other clusters were grouped together (“grey” module at the bottom in Fig 4B). There were very weak correlations between eigenvalue of “grey” module and each phenotypic trait, validating the accuracy of module clustering.

### Functional annotation enrichment analysis of gene cluster I

The genes from the three clusters of interest (*darkgreen*, *magenta* and *black*, identified above as being strongly correlated with phenotypic traits), were submitted to AgriGO [32] for Gene Ontology (GO) enrichment (S1 Fig). A total of 885 genes were grouped in cluster I (*darkgreen*). The eigengene for this cluster was positively correlated with stem growth and negatively correlated with PLH damage severity, suggesting that these genes are associated with plant growth and pest resistance. The eigengene expression profile (Fig 4C) for the parent genotypes revealed a trend of increasing expression over time after the pest infestation, and distinct differences in gene regulation, with induction of gene expression in the resistant parent 94006 versus suppression of expression in the susceptible parent ‘Jorr’. These distinct expression patterns suggest that the genes in this cluster are likely to be involved in defensive processes occurring in *S*. *purpurea* 94006.

Functional annotation of this set of genes includes significantly enriched GO terms in the biological process categories of ‘cell wall biosynthesis’ (GO:0042546, FDR = 1.9e^-14^), ‘aromatic compounds biosynthetic process’ (GO:0019438, FDR = 0.00027), ‘phenylpropanoid metabolic process’ (GO:0009698, FDR = 0.00059), and ‘response to stimulus’ (GO:0050896, FDR = 0.011) (S1 Fig). There are in total 15 enriched GO terms (FDR < 0.05) related to ‘cell wall biological process’ (GO:0042546, FDR = 1.7e^-6^). Specifically, the three top GO terms, ‘secondary cell wall biogenesis’ (GO:00009832, FDR = 1.3e^-9^), ‘xylan biosynthetic process’ (GO:0045492, FDR = 4.7e^-7^) and ‘hemicellulose metabolic synthesis’ (GO:0010410, FDR = 9.7e^-7^), highlight the role of the secondary cell wall metabolism, specifically cellulose, hemicellulose, and lignin synthesis, in plant defense strategies of the resistant genotype 94006. The ‘cellular aromatic compound metabolic process’ (GO:0006725, FDR = 0.00021) and ‘aromatic compounds biosynthetic process’ could also indicate lignin biosynthesis, since lignin is an aromatic polymer that mainly deposits during secondary cell wall thickening where it provides strength and rigidity. Secondary cell wall thickness in maize was found to be greater in genotypes resistant to obstruct mechanical rupture by corn borers within pith tissues [36]. In addition to lignin, phenylpropanoids and aromatic compounds and their derivatives are also precursors of the biosynthesis of flavonoids and condensed tannins, which have important biological functions in both abiotic and biotic stress defenses [37]. In addition, phenolic secondary metabolites play pivotal roles in plant chemical defense as antifeedants and toxins [38]. Finally, in cluster I, 105 genes were assigned to the ‘response to stimulus’ (GO:0050896; FDR = 0.011) biological process, indicating that PLH attack is perceived and signal transduction and basic defense responses are initiated in response to mechanical damage from chewing, or components of PLH saliva.

### Functional annotation enrichment of gene cluster II

There were 859 genes grouped in cluster II (*magenta*), for which the eigengene expression profile does not show a strong fluctuation over time after PLH feeding (Fig 4D). However, the expression of genes in cluster *magenta* in resistant genotype 94006 is constitutively greater compared to ‘Jorr’. The top most-represented GO terms in the biological process category was ‘response to stimulus’ (GO:0050896, FDR = 2.2e^-7^), which included 127 genes. Among the 127 response to stimulus genes, 51 were assigned to the child GO term ‘response to abiotic stimulus’ (GO:0009628, FDR = 0.001) and the other 53 were assigned to the two child GO terms ‘response to other organism’ (GO:0051707, FDR = 0.0051) and ‘response to biotic stimulus’ (GO:0009607, FDR = 0.0051) (S1B Fig). Among the other enriched GO terms in cluster II, some were associated with plant signaling perception and transduction, such as ‘hormone-mediated signaling pathway’ (GO:0009755, FDR = 9.1e^-5^) and ‘multidrug transport’ (GO:0006855, FDR = 7.8e^-5^). The Kyoto Encyclopedia of Genes and Genomes (KEGG) pathway mapping of cluster II genes identified the most enriched pathway as ‘tropane, piperidine and pyridine alkaloid biosynthesis’ (KO:00960, *p*-value = 0.0027). Alkaloids, derived from amino acids metabolism, are known to be anti-herbivory secondary metabolites. Production of various alkaloids suggests the host plant uses them as a chemical defensive barrier. Notably, three other KEGG pathways associated with signal transduction are also enriched: ‘other glycan degradation’ (KO:00511, *p*-value = 0.0127), ‘fatty acid degradation’ (KO:00071, *p*-value = 0.0159) and ‘ABC transporters’ (KO:02010, *p*-value = 0.031). Cell wall released free glycans act as signals that initiate plant defense response through recognition by receptors on plasma membrane [39]. Fatty acids (FAs) and their derived metabolites, which are released from membranes after triggered by environmental stimuli, function as second messengers and modulators of the plant innate immune system [40]. Plant ABC transporters contribute to the transportation of plant endogenous defensive secondary metabolites like alkaloids, terpenoids, polyphenols, quinones, etc. [41]. As well, fatty acid oxidation leads to the biosynthesis of jasmonic acid (JA) and salicylic acid (SA), two phytohormones vital in regulating plant defenses against biotic stress [42]. Furthermore, two Rad genes (Rad50, Rad27) were mapped to the KEGG pathway ‘non- homologous-end joining (NHEJ)’ (KO:03450, *p*-value = 0.022). Double strand DNA breaks (DSBs), which can be induced by both endogenous agents and environmental elicitors, are repaired mainly via the non-homologous end joining mechanism [43]. Studies detected DSBs in Arabidopsis after pathogen infections, which revealed the interconnection between DNA damage repair and plant immune resistance [44].

### Functional annotation enrichment of gene cluster III

Cluster III (*black*) includes 685 genes which were negatively correlated with stem growth and positively correlated with plant susceptibility (reflected as symptoms like necrosis, leaf curling and shoot damage). The eigengene expression pattern (Fig 4E) shows lower expression levels generally in the resistant parent 94006, but higher expression in the susceptible ‘Jorr’, as well as a general increase over time after PLH treatment. Gene ontology enrichment analysis for cluster 3 genes identified GO terms that are highly enriched in the biological process category of ‘death’ (GO:0016265, FDR = 2.9e^-8^), ‘cell death’ (GO:0008219, FDR = 2.9e^-8^), ‘cell programmed death’ (GO:0012501, FDR = 1.1e^-8^) and ‘apoptosis’ (GO:0006915, FDR = 1.7e^-6^), indicating these genes may account for pest damage symptoms such as necrosis, leaf curling and leaf abscission in the susceptible genotype due to programmed cell death in the leaves. Another enriched GO term, ‘defense response’ (GO:0006952, FDR = 1.3e^-5^), consists of 32 genes highly expressed in ‘Jorr’ specifically, but were expressed at low levels in 94006, indicating that the susceptible parent ‘Jorr’ had specific defensive responses after PLH attack that were insufficient to protect against PLH.

### PLH-resistance associated Transcription Factor (TF) genes and their regulatory networks

Transcriptional regulation of gene expression under stress conditions is pivotal to plant defense response [45]. Transcription factors (TFs) temporarily and spatially regulate the expression of their target genes via binding to *cis*-elements. To detect the master regulators within the resistance-related genes in the darkgreen cluster, the plant transcription factor database (PlnTFDB) [46] and PlantRegMap [47] were screened for regulation prediction and functional enrichment analyses via mapping the input genes to the curated Arabidopsis transcription regulatory interactions. Among the 885 genes in cluster I, 696 unique The Arabidopsis Information Resource (TAIR) IDs were assigned and 51 TFs in 18 families and 218 unique regulatory interactions were identified, forming the regulatory network shown in S2 Fig. Ten hub transcription factors in center of the network (assigned TAIR IDs: AT1G09540, AT1G32770, AT1G75240, AT1G78700, AT2G01940, AT3G12250, AT4G28500, AT4G29230, AT4G30080, AT5G12870) ranked highest for regulatory connectivity with other genes within this cluster (Table 1). Among the 10 hub genes, five genes are in the NAC (3) and MYB (2) families, associated with the secondary wall biosynthesis; four genes are involved in auxin (two ethylene and ARF TFs), abscisic acid (one ZF-HD TF) and brassinosteroid (one BES1 TF) signaling pathways; and the remaining one gene belongs to the bZIP family, which regulates plant systemic acquired resistance. The above functional annotation of the 10 TFs is in concordance with the results of GO and KEGG pathway enrichment analysis for the genes in this cluster, which highlights the defensive strategy for resistance in the parent genotype 94006 of several biotic resistance processes, including secondary cell wall strengthening, phytohormone signal transduction, and initiation of systemic acquired resistance.

**Table 1:** Functional annotation of 10 hub transcription factors in the WGCNA *darkgreen* cluster.

Hub Transcription Factor^α^	Transcription Factor Family	Functional Annotation ^β^
AT1G09540	MYB	vasculature development; xylem development
AT1G32770	NAC	lignin biosynthetic process; plant-type secondary cell wall biogenesis
AT4G28500	NAC	secondary cell wall biogenesis
AT4G29230	NAC	multicellular organism development; secondary cell wall biogenesis
AT2G01940	C2H2	auxin biosynthesis and transport
AT4G30080	ARF	auxin-activated signaling pathway; cell division
AT1G75240	ZF-HD	abscisic acid-activated signaling pathway
AT1G78700	BES1	brassinosteroid mediated signaling pathway
AT3G12250	bZIP	systemic acquired resistance
AT5G12870	MYB	defense response to fungus; plant-type secondary cell wall biogenesis

^α^: transferred TAIR ID.

^β^: functional ontology term summarized on Arabidopsis gene function description on TAIR database (https://www.arabidopsis.org).

### Pair-wise comparison of parents’ time-0 transcriptomes

**Fig 5:**
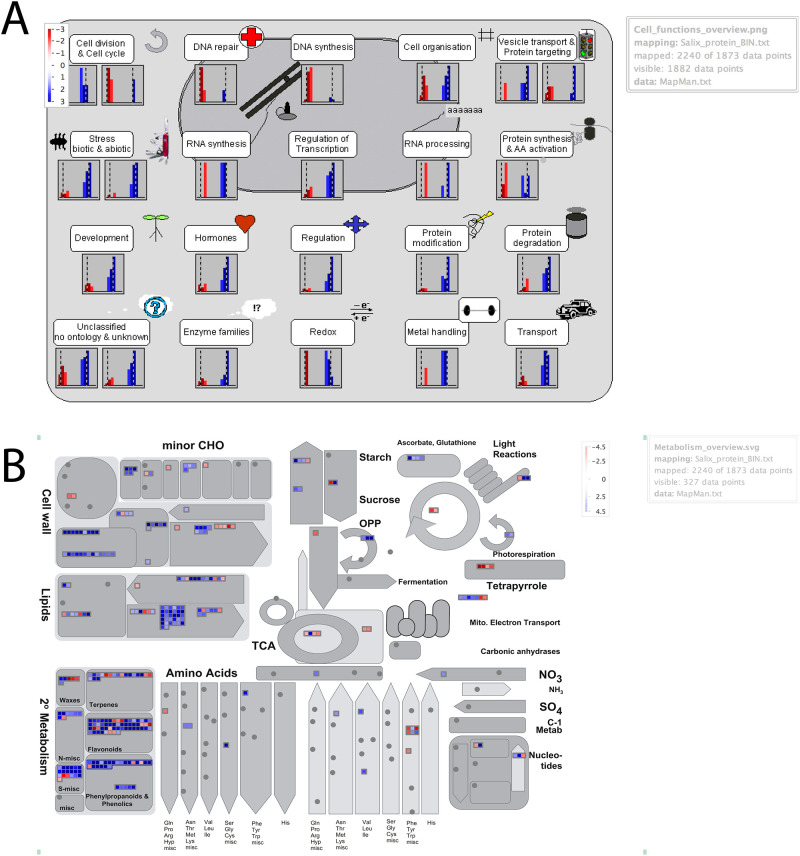
Overview distribution of log_2_ fold change of gene expression between parental species (*S*. *purpurea* vs. *S*. *viminalis*) by MapMan. (A) Cell function overview map shows the gene expression difference in various functional groups between *S*. *purpurea* and *S*. *viminalis*. Color coded bars represent the ratio of gene expression in *S*. *purpurea* vs. *S*. *viminalis* (Red, higher levels in *S*. *purpurea*; blue, higher levels in *S*. *viminalis*). The intensity of the color change corresponds to log2 fold change. (B) On the metabolism overview map, each BIN or subBIN is represented as a block where each gene is displayed as a square which is either colored blue if this gene is highly expressed in *S*. *purpurea* or red if this gene is highly expressed in *S*. *viminalis*. Metabolites displayed as circles and proteins displayed as triangles. Note: only ratios with *p*-values lower or equal to 0.05 are displayed.

There was a remarkable discrepancy between the greenhouse no- choice feeding experiment and the field trial. The resistant genotype 94006 displayed significantly less damage than susceptible ‘Jorr’ in the field, as expected. However, the greenhouse no-choice feeding trial showed that PLH also caused characteristic damage when forced to feed on the genotypes that were resistant in the field, which suggests that host-choice by the PLH adults might be the primary basis for field resistance of *S*. *purpurea*. To test the hypothesis of host-choice as a resistance mechanism in genotype 94006, a comparison of the parents’ time 0 transcriptomes, prior to challenge by PLH, was conducted. In the time 0 samples, the genes that were most highly expressed in the susceptible ‘Jorr’ genotype (*S*. *viminalis*) were significantly enriched in the GO terms ‘cellular process’ (GO:0009987, FDR = 1.8e^-27^) and ‘metabolic process’ (GO:0008152, FDR = 3.4e^-20^) (Table 2). In contrast, the resistant species *S*. *purpurea* parent harbored genes with constitutively higher expression levels, relative to *S*. *viminalis* parent, that were assigned to the top GO term ‘response to stimulus’ (GO:0050896, FDR = 2e^-22^), which includes biotic stress response sub-categories such as ‘response to biotic stimulus’ (GO:0009607, FDR = 8.6e^-8^) and ‘response to chitin’ (GO:0010200, FDR = 2.3e^-7^), which were highly expressed (Table 2). In addition to GO term enrichment analysis, MapMan [34] was used, another functional annotation software, to identify and visualize the processes and pathways distinctly enriched between two parent species (Fig 5). Fig 5A shows the overall mapping of DEG in different functional groups as categorized within MapMan and Fig 5B highlights the relevant transcriptional changes related to overall metabolism. Consistent with the GO analysis, the highly expressed genes in *S*. *purpurea* mapped primarily to functional categories of biotic and abiotic stress (Fig 5A). As expected, genes highly expressed in *S*. *viminalis* mapped to categories of protein synthesis and amino acid activation and cell division and cell cycle. Another interesting finding was a group of genes with greater expression in *S*. *viminalis* that were assigned to DNA repair and DNA synthesis processes, which may relate to faster vegetative growth in this parent. A closer look at the detailed metabolic pathways showed that genes with greater expression in *S*. *viminalis* were mapped to photosynthesis metabolism, especially the photorespiration process, which again may be related to vigorous growth. The genes expressed more highly in *S*. *purpurea* were largely involved in the biosynthesis of secondary metabolites such as terpenes, flavonoids, phenylpropanoids and phenolics. These metabolites have roles as toxins and feeding deterrents which play important defensive roles against many herbivorous insects [48].

**Table 2:** GO term enrichment of DEGs between transcriptomes of parent genotypes *S*. *purpurea* 94006 and *S*. *viminalis* ‘Jorr’ at time 0.

Resistant *S*. *purpurea*		Susceptible *S*. *viminalis*	
*Gene Functional Ontology*	*FDR* ^*a*^	*Gene Functional Ontology*	*FDR* ^*a*^
response to stimulus	2e^-22^	cellular process	1.8e^-27^
response to chemical stimulus	1.1e^-14^	metabolic process	3.4e^-20^
response to organic substance	3.2e^-11^	primary metabolic process	8.4e^-17^
response to stress	1.2e^-09^	cellular metabolic process	8.4e^-17^
response to biotic stimulus	8.6e^-08^	biosynthetic process	8.6e^-17^
response to chitin	2.3e^-07^	cellular biosynthetic process	1.9e^-16^

^α^ False discovery rate (α = 0.05).

### Dominance accounts substantially for differential expression among F_1_ progeny

**Fig 6:**
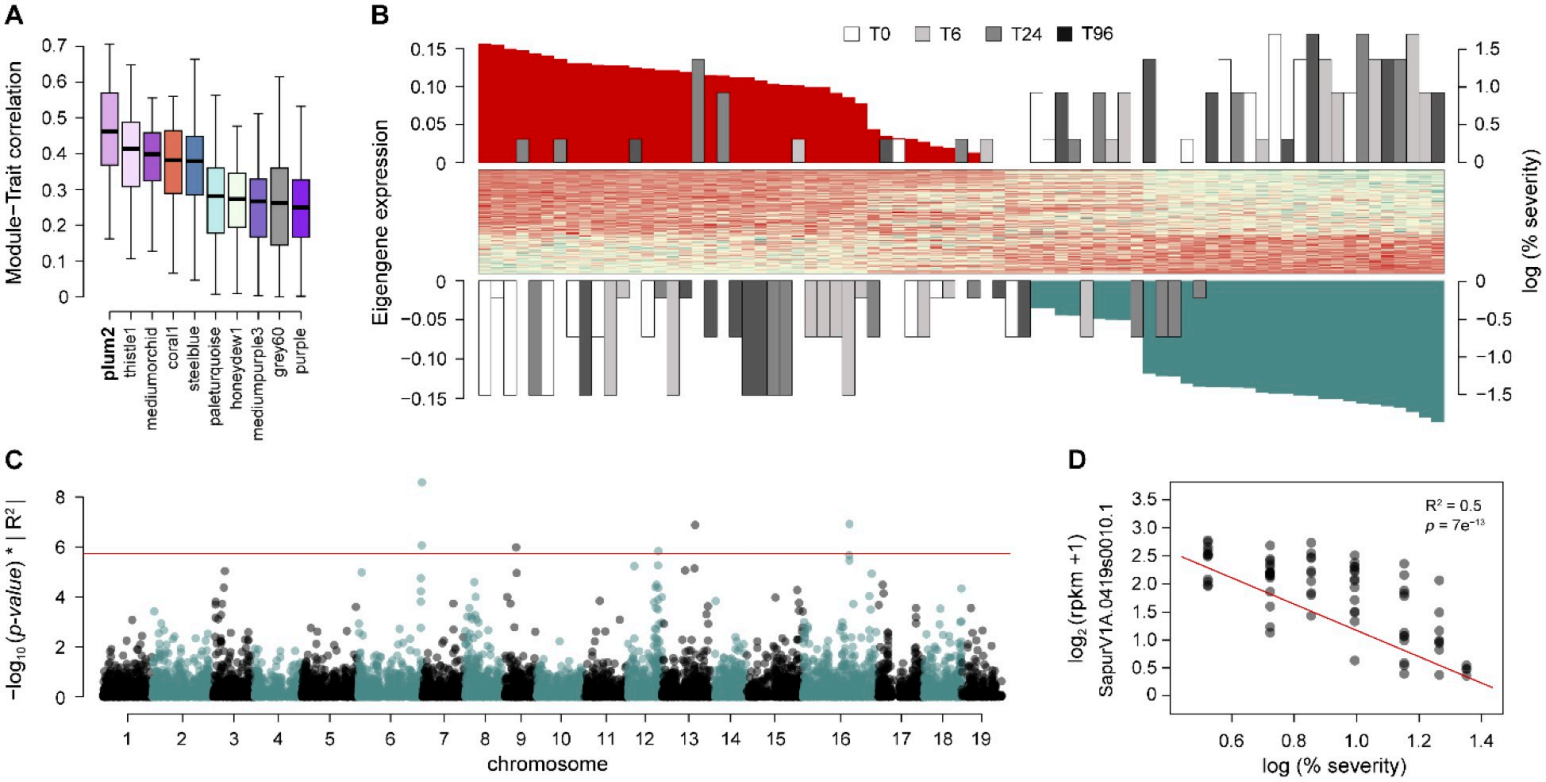
Chromosome-wide patterns of inheritance of gene expression in F_1_ *S*. *purpurea* × *S*. *viminalis* progeny individuals. For each of the 19 chromosomes of *Salix*, the middle black bar separates T0 (left) from T6 (right). An equal number of bins (n = 12), ordered by their physical position, represents the total number of genes in each inheritance class (scale, top left). Inheritance classifications are stacked within each bin and are colored according to the legend (center top).

The mode of gene expression inheritance was assessed in F_1_ interspecific S. *purpurea* × S. *viminalis* progeny at the four different time-points. For each time-point, gene expression inheritance patterns of the F_1_ individuals were relatively uniform across the 19 haploid chromosomes (Fig 6). By comparing samples across time-points, there were significant differences in the number of DEG and patterns of gene expression inheritance. Overall, the average number of P1 (*S*. *purpurea*) dominant genes of F_1_ family declined over the 96 h period, in a linear decreasing fashion, from 1552 at T0 to 988 at T96 (S1 Table). However, the average number of P2 (*S*. *viminalis*)—dominant genes increased from 1125 at T0 to 1485 at T96 (S1 Table). Transgressive gene expression increased from T0 to T96 but was less dramatic compared to those showing uniparental or dominant patterns of inheritance, whereas the converse was found for genes with additive expression inheritance on average (70 at T0 and 58 at T96). In a previous study, using full-sib intraspecific F_1_ and F_2_ *S*. *purpurea* families, Carlson [49] described dominant inheritance as the primary source of differential expression in both shoot tip and internode tissues. While there was less differential expression in the F_2_ family, the F_1_ S. *purpurea* individuals showed a high proportion of maternal P1-dominant gene expression, irrespective of the tissue type. Likewise, dominance accounts for a large majority of the differential expression identified in the F_1_ S. *purpurea* x S. *viminalis* family. However, over a seemingly brief period of time (96 hrs), P1:P2 ratios of uniparental inheritance among the F_1_ individuals were not static, but tended to oscillate (S3 Fig).

### Real-time quantitative PCR analysis of DEGs in parent genotypes

To confirm that the RNA-Seq profiles accurately reflected the actual expression levels for each sample, six genes encoding four transcription factors (TFs) (SapurV1A.1016s0030, MYB; SapurV1A.0106s0050 Sgf11; SapurV1A.0534s0230, MYB2; SapurV1A.2717s0010, NAC), 1 ligandins (SapurV1A.0427s0070, Glu_b) and one heat shock protein (SapurV1A.0571s0080, FBK) in both willow genotypes, 94006 and ‘Jorr’, were selected for quantitative real-time PCR analysis. The primers used in the qPCR are listed in S2 Table. These genes were chosen for their contrasting expression patterns in response to PLH feeding between the two parent genotypes. The membrane-anchored ubiquitin-fold protein, MUB (SapurV1A.2454s0040), was used as the endogenous reference to normalize all qPCR measurement. The log-fold change of each qPCR measurement was compared to the log-fold change of RNA-Seq normalized value and displayed side by side as bar chart (S3 Table and S4A Fig). The regression analysis revealed a significant positive correlation (Pearson *r* = 0.93) in the expression profiles between RNA-seq and qPCR data (S4B Fig), confirming that there were not any significant biases in the RNASeq data from technical issues in the RNA isolation and sequencing process.

## Discussion

### Biosynthesis of secondary cell wall compounds as a compensation for PLH injury

The ability to recover from the insect damage largely determines the resistance or tolerance of a plant under herbivore stress. The sooner the plant deploys an effective recovery, the less damage accumulates from pest feeding. Previous studies revealed that PLH employ a lacerate-and-flush feeding behavior [13, 17], which consists of rupturing cells by rapid movement of their stylets, causing mechanical damage, and simultaneously salivating and withdrawing phloem liquid. Herbivory by PLH induces a cascade of anatomical and physiological disturbances in alfalfa, their primary host: injured phloem tissues first suffer cell wall loosening and collapse, followed by phloem blockage [50, 51]. The characteristic symptom “hopperburn” is largely the result of small blockages and phloem constrictions in the plant vasculature. The following recovery phase is the regeneration of wound phloem sieve elements that circumvent the damaged phloem cells [13, 16, 52] as a result, xylem tissues are reduced in size and quantity, and the mature tracheary elements are compensated for by generation of numerous, thick-walled sclerenchyma fiber cells.

Here, the resistant *S*. *purpurea* 94006 genotype rapidly induced an arsenal of genes (within 6 h of PLH feeding) sharing the pattern of increased expression levels coinciding with the pest feeding, attaining much higher levels than in susceptible genotypes in which defense genes were induced more gradually. This unique expression pattern suggests that these genes are involved in the effective defensive processes in the resistant 94006 genotype. Functional annotations illustrate their roles in cell wall biosynthesis, especially those components abundant in secondary cell wall (xylan, glucuronoxylan, and lignin). Our transcriptome results are consistent with phenotypic observations in which PLH first injure the vascular tissue, followed by host recovery that depends largely on the generation of new vascular transport cells to restore translocation function. In the resistant genotype 94006, the biosynthesis of plant cell walls (especially secondary cell walls) could both restore cell wall integrity and reinforce the secondary cell walls by increasing cell wall thickness. Our observations with *S*. *purpurea* 94006 were similar to examples reported in maize, in which increased secondary cell wall thickness prevents mechanical rupture of the pith tissues caused by corn borer insect feeding [36], while re-differentiation of tracheids and sieve elements with thick lignified secondary cell walls restored the flow of nutrients and water to maintain normal physiological activities.

### Different strategies for plant growth, constitutive and induced resistance between species

Plants produce limited photosynthates and acquire other resources which are essential for both plant growth and defense [53]. Developing defensive traits comes at the cost of reduced growth and reproduction [54]. For example, it was observed in *Nicotiana* that increased defense was usually accompanied by attenuated growth rate [55]. During their evolution, species occupying divergent environments developed various strategies to better adapt to specialized habitat and to balance the growth-defense trade-offs [56, 57]. *Salix viminalis* and its hybrids represent the most widely grown willow cultivars as a short rotation crop in Europe [58, 59] and is favored for its multiple desirable traits in aspect of biomass production, including high yield, fast growth, good coppicing, and good maintenance of growth form [60]. Even with high sensitivities to pests and diseases, *S*. *viminalis* is still popular in breeding programs as hybridized with other *Salix* species to take advantage of its vigorous growth traits in the F_1_ hybrid [22, 61]. In our study, by comparing the transcriptomes of both *S*. *viminalis* and *S*. *purpurea* in the absence of herbivory, we identified the genes associated with cellular metabolism and biosynthesis were more highly expressed in susceptible species *S*. *viminalis*, indicating its rigorous growth status. On the other hand, the resistant species *S*. *purpurea* showed greater expression of genes involved in stress and stimulus response, combined with morphological trait of smaller leaves and lower yield in field trials, suggesting diverted resources from growth to constitutive resistance in *S*. *purpurea*, compared with *S*. *viminalis*. Whereas in presence of herbivory, *S*. *purpurea* promptly initiated induced resistance within 6 h, as we identified a group of genes (Gene cluster I in WGCNA analysis) contributing to secondary cell wall thickening were increasingly expressed after herbivory imposing. Thus, despite the constitutive resistance, *S*. *purpurea* also rapidly induced structural defensive traits, which brings a fitness advantage by direct discouragement of herbivore feeding [62], and fast restoration of damaged tissue. While in *S*. *viminalis*, another induced defense strategy dominated. Within 24 h after herbivory, a set of genes (Gene cluster III in WGCNA analysis) involved in program cell death upregulated in response to PLH herbivory in *S*. *viminalis*. This programmed cell death defensive response is called a hypersensitive response (HR) and prevails in plant defense against pathogens [63, 64]. Ollerstam [65] reported HR-like response in *S*. *viminalis* leaves within 12 h of gall midges egg hatch and identified a strong association with larval mortality. While the plant HR defensive response is primarily implicated with herbivores in sessile life stage, it is obviously not an effective defense against actively moving herbivore like PLH adults. Additionally, even though induced resistance is cost-saving compared to constitutive resistance [66], time-lags before induced resistance taken into action may compromise the effectiveness and cause irreversible damage [67]. In summary, interspecies diverse strategies are deployed to balance the trade-offs among plant growth, constitutive and induced defense, under current environmental context where the plants grow.

### High correlations between NBS-LRR genes and PLH resistance and sex

**Fig 7:**
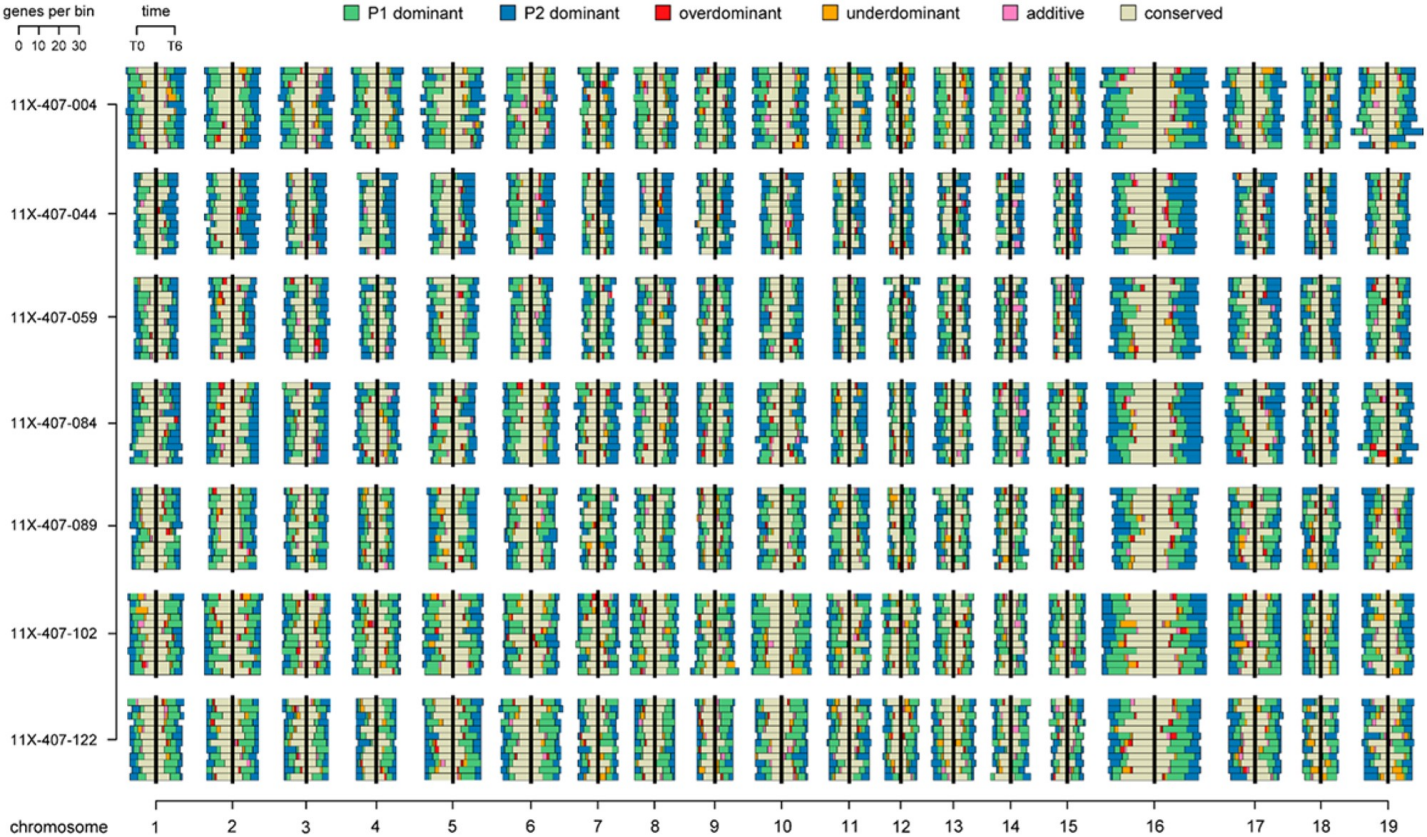
Co-expression analyses of F_1_ *S*. *purpurea* × S. *viminalis* progeny and Panel A. Boxplots represent the significance of eigengene-trait relationship correlations between pest damage severity and 10 co-expressed gene modules with highest correlation to the percent severity of the pest damage trait. The *plum2* module had the highest module-trait correlation. Panel B. The scaled eigengene expression heatmap for the *plum2* module and scaled log percent severity of damage trait values are ordered according to their respective module membership (correlation between each gene and eigengene). The time of collection for each sample time-point 0, 6, 24, and 96 hr (top), are represented by white, light grey, dark grey, and *black* boxes, respectively. Panel C. The Manhattan plot depicts genome-wide significance for log damage severity (%). Gene models were aligned to the *S*. *purpurea* 94006 v1 reference assembly. The absolute R^2^ multiplied by the corresponding −log_10_ (*p*-value) (y-axis) is plotted against the physical position (Mb) of each gene (x-axis). The horizontal red line represents the genome-wise Bonferroni significance threshold, −log_10_ (*p*-value = 0.05/*n*). Panel D. The scatterplot shows the regression of log percent severity and the normalized expression of the *S*. *purpurea* NBS-LRR gene, *SapurV1A*.*0419s0010*.*1* (R^2^ = 0.5, *p*-value = 7e^-13^).

Resistance genes (R genes) are ubiquitous in land plants, especially those encoding nucleotide binding site-leucine-rich repeat (NBS-LRR) genes [68, 69], and have been shown to contribute to host resistance to pathogens in many genera [70]. In general, NBS domains have highly conserved motifs (e.g., P-loop, kinase-2, and Gly-Leu-Pro-Leu motifs), which are involved in protein-protein interactions. Toll interleukin repeat (TIR)- and non-TIR-NBS-LRRs are highly abundant in the reference genomes of both *S*. *purpurea* and *S*. *viminalis*, as well as that of a close relative, *Populus trichocarpa* [71]. Over 400 NBS-LRRs members of this large R gene family have been annotated in the *S*. *purpurea* v1.0 reference transcriptome assembly, a family size similar to that of *P*. *trichocarpa* [72]. In our study, genes significantly correlated (*p*-value < 1e^-6^) with the PLH severity phenotype in F_1_ plants were enriched for the NBS-LRR R gene family. Based on the pest symptom severity, the F_1_ progeny were categorized as susceptible or resistant (less susceptible) plants. In general, the TIR clade of NBS-LRRs was highly-expressed in susceptible plants, but coiled-coil (CC) NBS-LRR clade was more abundant in resistant or less-susceptible individuals. Also, P2-dominant patterns of inheritance were over-represented for TIR-NBS-LRR genes, whereas P1-dominant patterns were over-represented for genes in the CC-NBS-LRRs clade. Divergent evolution of NBS-LRR R genes between *S*. *purpurea* and *S*. *viminalis* might account for these striking patterns of uniparental dominant gene expression and PLH resistance among their progeny. Co-expression analyses of F_1_ progeny clustered a large proportion of NBS-LRR gene family members into modules that were significantly correlated to resistance traits observed both in the greenhouse and in the field. The *plum2* module showed the greatest correlation to the log PLH severity (%) in F_1_ progeny (Fig 7A), compared to all other modules. Moreover, for a majority of genes within the *plum2* module, there was a strong negative correlation with log PLH severity (Fig 7B). Among them are eight genes identified possessing NBS-LRR motifs (Fig 7C). SapurV1A.0419s0010, a gene with the greatest inverse correlation for log PLH severity (%), was highly-expressed in resistant and less-susceptible plants (Fig 7D), and annotated as an apoptotic CC-NBS-LRR gene, homologous to Arabidopsis receptor kinase ZED1. In Arabidopsis, *ZED1* is predicted to act as a decoy for the *Pseudomonas syringae* HopZ1 effector acetyltransferase [73] which is required for recognition by the R gene, *ZAR1*, such that mutant plants lacking *ZED1* showed increased pathogen growth.

Another NBS-LRR clade, called the BED finger NBS-LRRs, were identified as highly correlated with progeny sex (R^2^ = 0.75 − 0.99, *p*-value < 1e^-60^), and are primarily concentrated on *S*. *purpurea* chr15 and chr19 (S5 Fig). The tandem duplication of NBS-LRR R genes is common in land plants and they tend to cluster over time. The three most significant BED finger NBS-LRRs located near the end of chr17 (SapurV1A.1003s0080, SapurV1A.1003s0090, and SapurV1A.1379s0010), share common domain architecture and are in relatively close proximity to one another (~10 Kb). The BED finger NBS-LRR, SapurV1A.1005s0080, with the greatest correlation with sex (R^2^ = 0.99, *p*-value = 1e^-75^), is located in a peritelomeric region of *S*. *purpurea* chr19, a region orthologous to the peritelomeric sex determining region (SDR) on *P*. *trichocarpa* chr19. Sex quantitative trait loci (QTL) have been mapped on chr15 in full-sib F_1_ and F_2_ biparental families and association panels of both *S*. *purpurea* [5, 49] and *S*. *viminalis* [74, 75]. Difference in gene content along the *S*. *purpurea* chr15 SDR or the putative ancestral Y chromosome (chr19) is likely to be more pronounced when comparing gene expression levels of plants of opposite sex undergoing biotic stress, especially for genes within regions of low recombination.

In order to breed new cultivars with durable resistance to PLH under increasingly warmer climate conditions, it is critical to understand the genetic basis for resistance and how it can be introgressed into improved cultivars. We have identified differentially expressed genes that are potentially relevant in determining functional susceptibility to PLH infestation. These genes can be targeted to inform decisions in choosing parents of hybrid crosses and the early selection of potentially resistant individuals for cultivar development. The identification of loci influencing the expression patterns of these candidate resistance genes (eQTL) can also be the focus of molecular marker development to facilitate the efficient early selection of parents for crosses and progeny compared to field trial screening, which is far more laborious and costly. We aim to validate these potential candidate genes across pedigrees, then incorporate a molecular breeding strategy toward long-term improvement of high-yielding cultivars.

## Supporting information

S1 Fig Hierarchical tree graphs of over-represented GO (gene oncology) terms in biological process categories for co-expressed genes in the *darkgreen*, *magenta* and *black* modules by singular enrichment analysis generated by AgriGO. Each box represents a GO term in biological process category, labeled with the GO term ID, term definition. The significantly enriched GO terms were identified by threshold of FDR ≤ 0.05 (FDR value shown in the brackets after term id), and filled with red-yellow colors, while non-significant terms are shown as white boxes. The degree of color saturation of a box is positively correlated to the significance level of the term. The color and type of lines represent different regulatory relationships (elaborated in legend window). The hierarchical rank of GO term decreases from top to bottom.
(TIF)

S2 Fig The internal regulations and connectivity network among genes in the *darkgreen* co-expressed gene cluster. The 10 hub transcriptional factors (AT1G09540, AT1G32770, AT1G75240, AT1G78700, AT2G01940, AT3G12250, AT4G28500, AT4G29230, AT4G30080, AT5G12870) are highlighted in yellow, which have the highest regulatory connectivity with other genes within this cluster. Green arrow: negative regulation; red arrow: positive regulation.
(TIF)

S3 Fig Distribution of inheritance classes of genes among F_1_ *S*. *purpurea* × *S*. *viminalis* progeny individuals. Left column, Scatter plots of classes of gene expression inheritance patterns. Center column: Bar charts of same data for classes of gene expression inheritance patterns. Right column: Bar charts of inheritance patterns for P1>P2 vs. P2>P1. Replicates for genes were summed for each time point; Genes were filtered (CPM > 0.5, ≥ 30%) and assigned inheritance classifications for only those with significant DE (FDR = 0.05).
(TIF)

S4 Fig Transcript abundances of six genes from 43 samples were measured by both RNA-Seq and qPCR. Panel (A) horizontally listed the fold change values of each gene across time within both genotypes, measured by both RNA-Seq data (red column) and qPCR data (blue column) and compared side by side in the bar chart. Panel (B) shows the high correlation (R^2^ = 0.93) of the log fold change between RNA-Seq (y axis) and qPCR (x axis).
(TIF)

S5 Fig Manhattan plot of genome-wide distribution of significance of sex-biased gene expression in F_1_ *S*. *purpurea* × *S*. *viminalis* progeny individuals. The absolute R^2^ multiplied by the corresponding −log_10_ (*p*-value) (y-axis) is plotted against the physical position (Mb) of each gene (x-axis). The horizontal red line represents the genome-wise Bonferroni significance threshold, −log_10_ (*p* = 0.05/*n*).
(TIF)

S1 Table Inheritance patterns of global gene expression among all F_1_ *S*. *purpurea* × *S*. *viminalis* progeny individuals. Rows are ordered by time, starting with the control time point (T0), then numerically by clone identifiers. For each time-point, the total number of genes belonging to inheritance classes was summarized by the family average.
(DOCX)

S2 Table Primer information for qPCR validation of selected genes. (DOCX)

S3 Table Validation of differentially expressed genes related to transcription factors (TFs), ligandins and heat shock protein of two contrasting PLH-susceptible shrub willow genotypes. The table also illustrates the functional characteristics of each genes.
(DOCX)
