# Supplementary figures and images for: Transcriptome analysis of contrasting resistance to herbivory by Empoasca fabae in two shrub willow species and their hybrid progeny
Source: PLoS One. 2020 Jul 29;15(7):e0236586. doi: 10.1371/journal.pone.0236586 (PMC7390382; doi:10.1371/journal.pone.0236586)

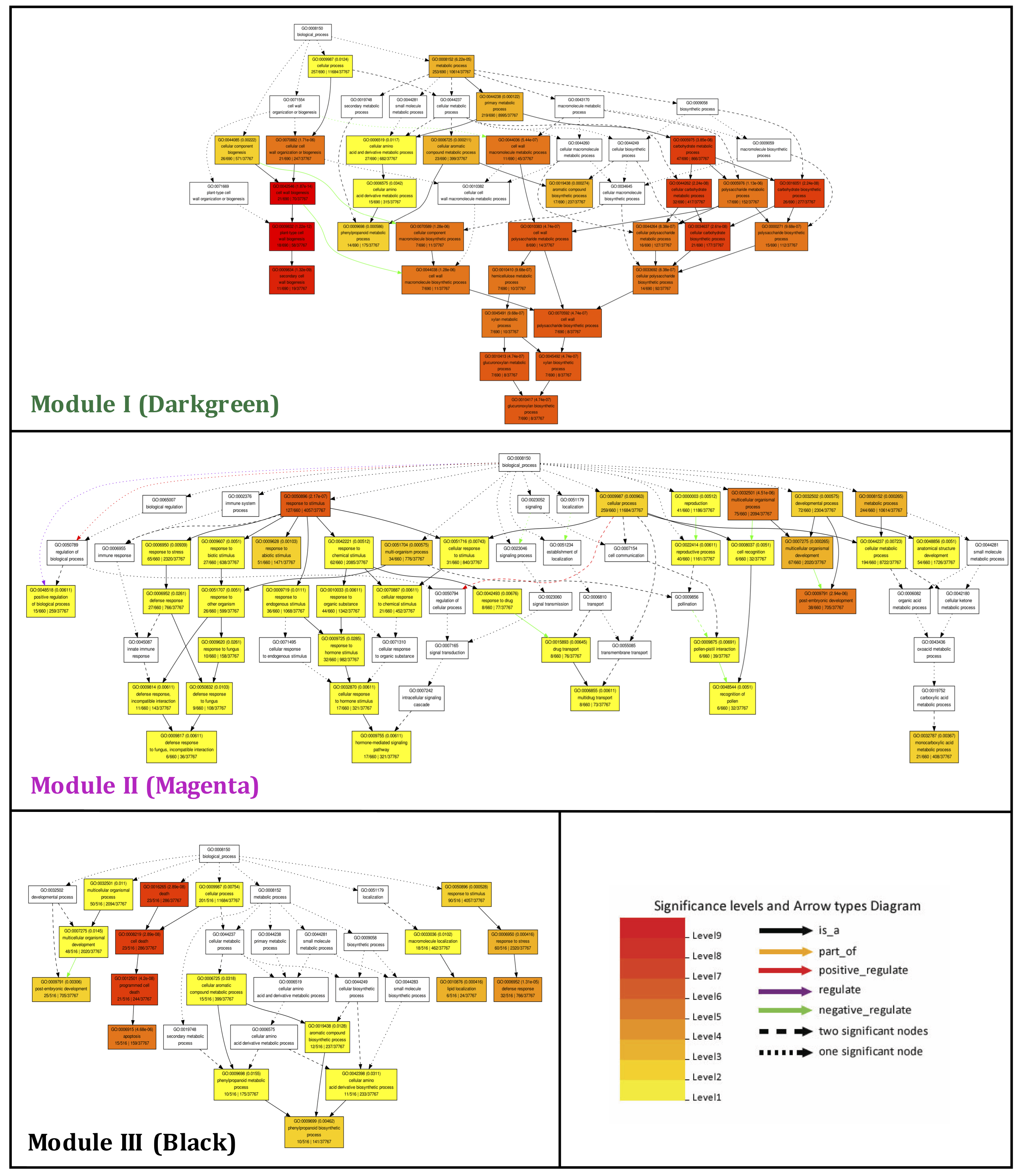

Supplement: S1 Fig — Each box represents a GO term in biological process category, labeled with the GO term ID, term definition. The significantly enriched GO terms were identified by threshold of FDR ≤ 0.05 (FDR value shown in the brackets after term id), and filled with red-yellow colors, while non-significant terms are shown as white boxes. The degree of color saturation of a box is positively correlated to the significance level of the term. The color and type of lines represent different regulatory relationships (elaborated in legend window). The hierarchical rank of GO term decreases from top to bottom. (TIF) [file pone.0236586.s001.tif]

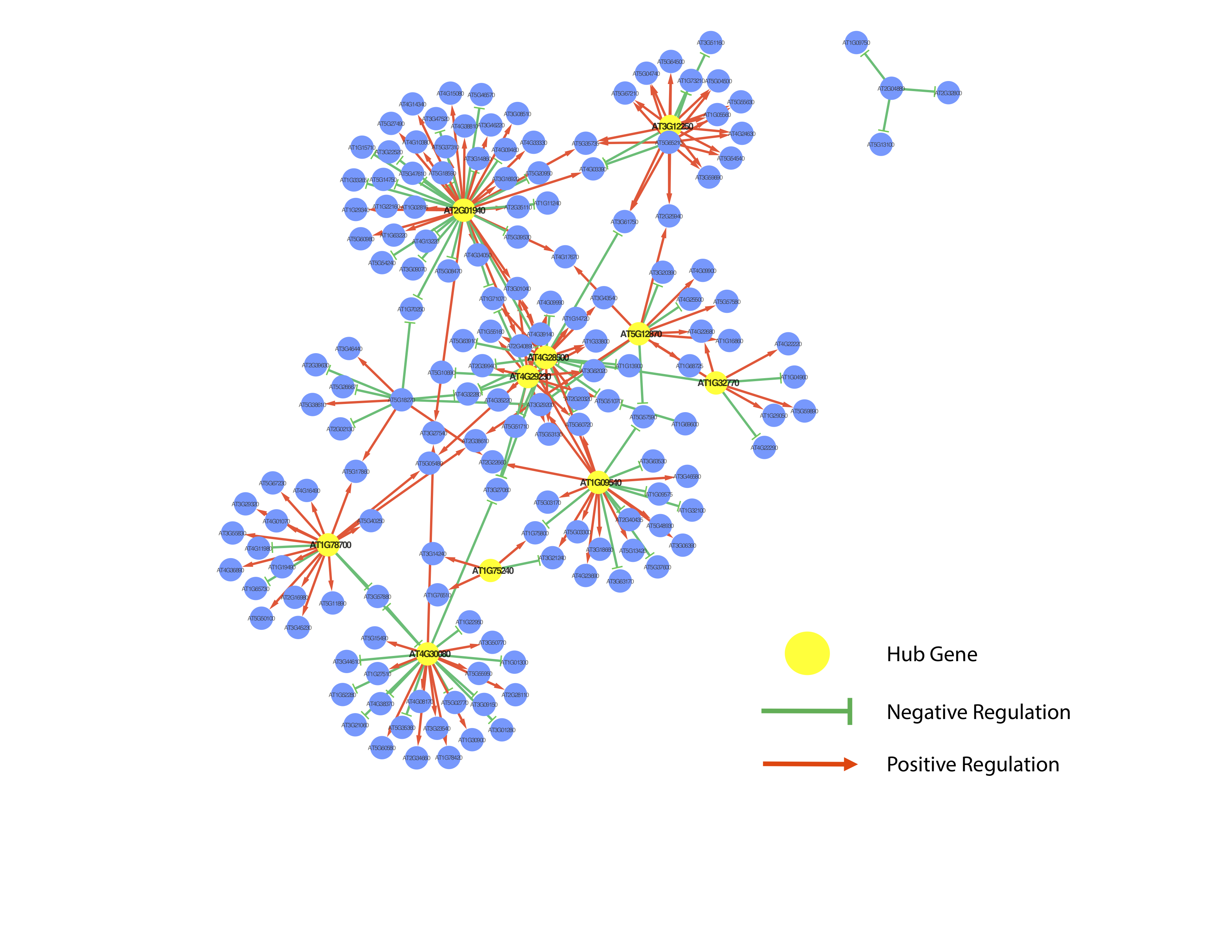

Supplement: S2 Fig — The 10 hub transcriptional factors (AT1G09540, AT1G32770, AT1G75240, AT1G78700, AT2G01940, AT3G12250, AT4G28500, AT4G29230, AT4G30080, AT5G12870) are highlighted in yellow, which have the highest regulatory connectivity with other genes within this cluster. Green arrow: negative regulation; red arrow: positive regulation. (TIF) [file pone.0236586.s002.tif]

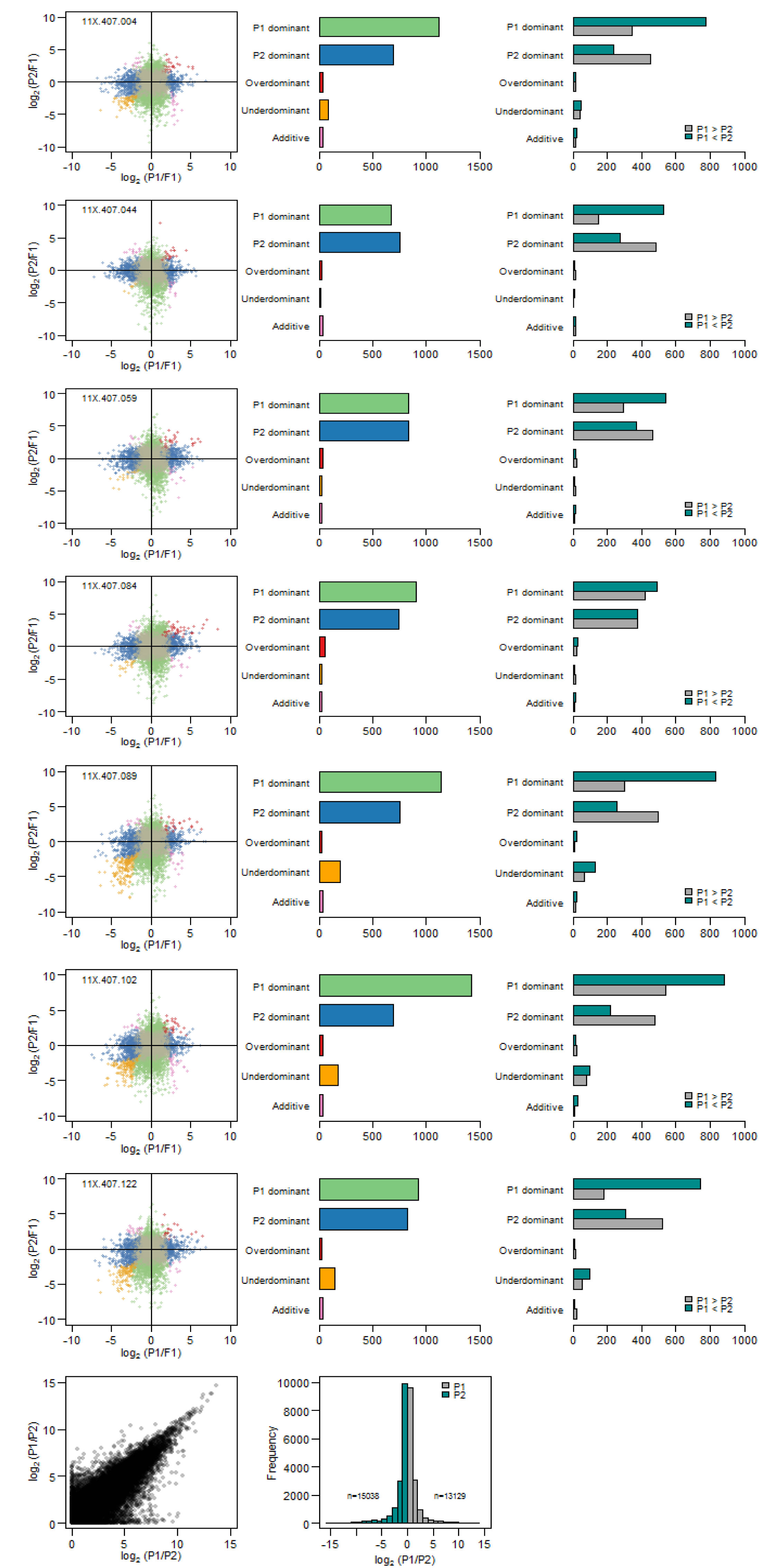

Supplement: S3 Fig — Left column, Scatter plots of classes of gene expression inheritance patterns. Center column: Bar charts of same data for classes of gene expression inheritance patterns. Right column: Bar charts of inheritance patterns for P1>P2 vs. P2>P1. Replicates for genes were summed for each time point; Genes were filtered (CPM > 0.5, ≥ 30%) and assigned inheritance classifications for only those with significant DE (FDR = 0.05). (TIF) [file pone.0236586.s003.tif]

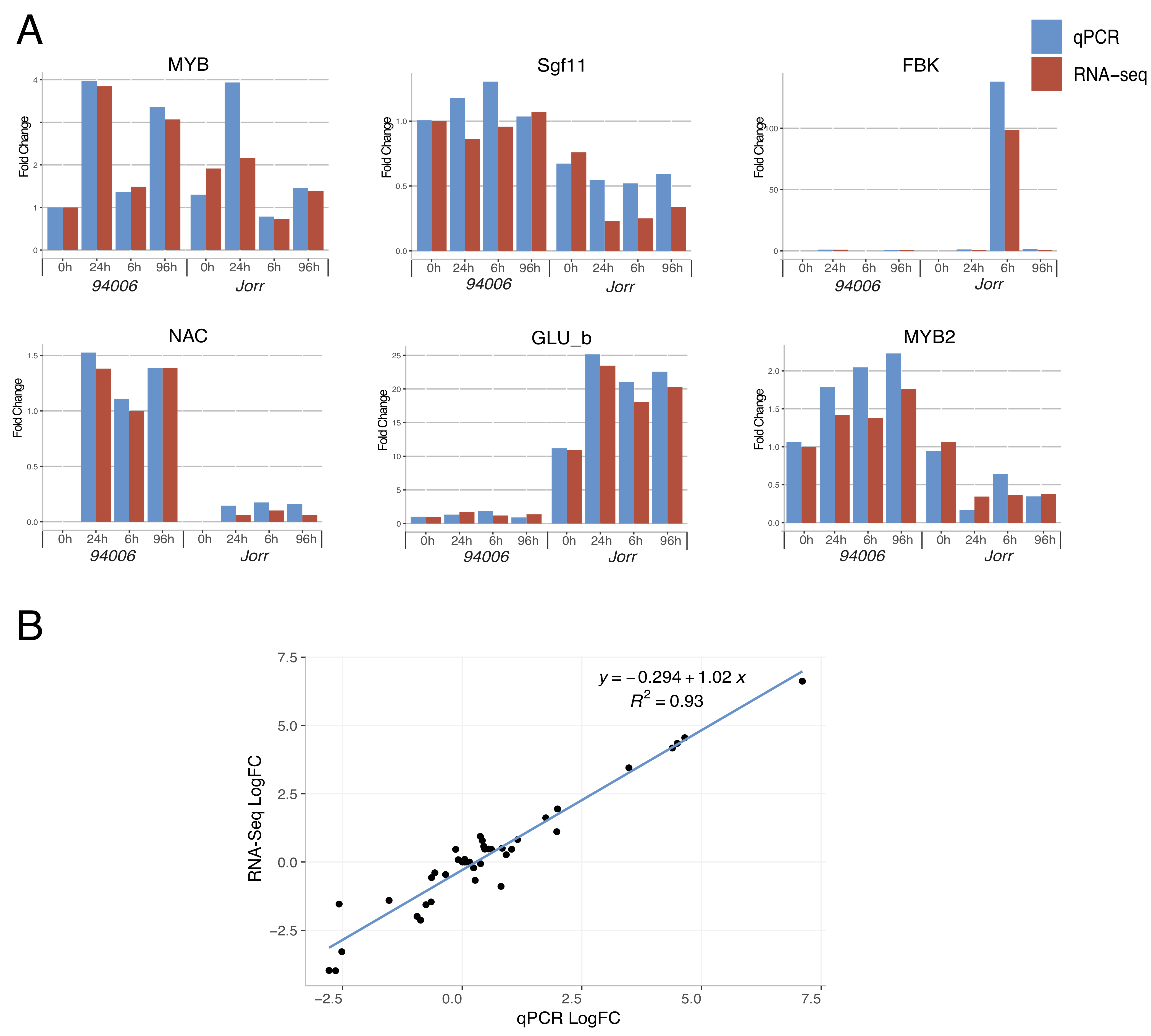

Supplement: S4 Fig — Panel (A) horizontally listed the fold change values of each gene across time within both genotypes, measured by both RNA-Seq data (red column) and qPCR data (blue column) and compared side by side in the bar chart. Panel (B) shows the high correlation (R2 = 0.93) of the log fold change between RNA-Seq (y axis) and qPCR (x axis). (TIF) [file pone.0236586.s004.tif]

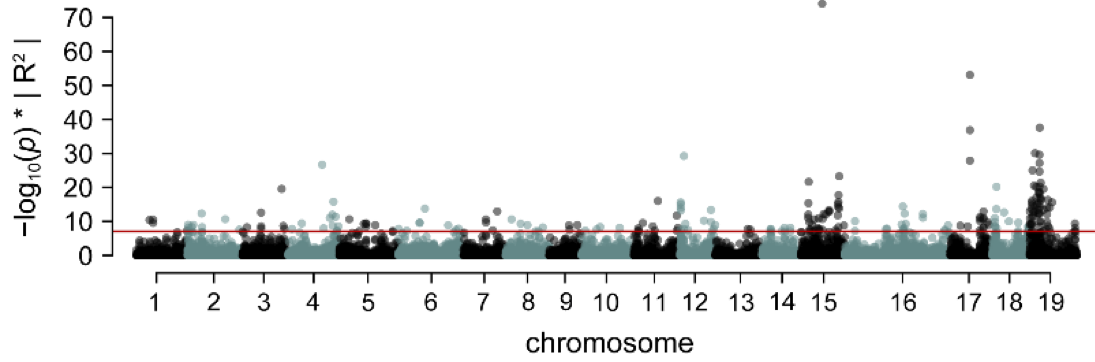

Supplement: S5 Fig — The absolute R2 multiplied by the corresponding −log10 (p-value) (y-axis) is plotted against the physical position (Mb) of each gene (x-axis). The horizontal red line represents the genome-wise Bonferroni significance threshold, −log10 (p = 0.05/n). (TIF) [file pone.0236586.s005.tif]
