# Supplementary material for: Transcriptome analysis of contrasting resistance to herbivory by Empoasca fabae in two shrub willow species and their hybrid progeny
Source: PLoS One. 2020 Jul 29;15(7):e0236586. doi: 10.1371/journal.pone.0236586 (PMC7390382; doi:10.1371/journal.pone.0236586)
Supplement: S1 Table — Rows are ordered by time, starting with the control time point (T0), then numerically by clone identifiers. For each time-point, the total number of genes belonging to inheritance classes was summarized by the family average. (DOCX) [file pone.0236586.s006.docx]

**S1 Table. Inheritance patterns of global gene expression among all F_1_ *S. purpurea* × *S. viminalis* progeny individuals.** Rows are ordered by time, starting with the control time point (T0), then numerically by clone identifiers. For each time-point, the total number of genes belonging to inheritance classes was summarized by the family average.

| Clone ID | | Time | | P1-dominant | | P2-dominant | | Over dominant | | Underdominant | | Additive | | Conserved |
| --- | --- | --- | --- | --- | --- | --- | --- | --- | --- | --- | --- | --- | --- | --- |
| 11X-407-004 | | 0 | | 1781 | | 1045 | | 52 | | 156 | | 85 | | 27332 |
| 11X-407-044 | | 0 | | 1001 | | 1198 | | 46 | | 19 | | 78 | | 28109 |
| 11X-407-059 | | 0 | | 1316 | | 1176 | | 58 | | 40 | | 59 | | 27802 |
| 11X-407-084 | | 0 | | 1366 | | 1155 | | 119 | | 46 | | 51 | | 27714 |
| 11X-407-089 | | 0 | | 1719 | | 1065 | | 54 | | 288 | | 64 | | 27261 |
| 11X-407-102 | | 0 | | 2274 | | 1002 | | 73 | | 283 | | 86 | | 26733 |
| 11X-407-122 | | 0 | | 1404 | | 1236 | | 61 | | 242 | | 69 | | 27439 |
| 11X-407-004 | | 6 | | 1395 | | 1508 | | 43 | | 276 | | 100 | | 27129 |
| 11X-407-044 | | 6 | | 775 | | 1916 | | 81 | | 22 | | 98 | | 27559 |
| 11X-407-059 | | 6 | | 1081 | | 1503 | | 59 | | 21 | | 77 | | 27710 |
| 11X-407-084 | | 6 | | 1048 | | 1526 | | 66 | | 69 | | 89 | | 27653 |
| 11X-407-089 | | 6 | | 1958 | | 1119 | | 59 | | 210 | | 79 | | 27026 |
| 11X-407-102 | | 6 | | 2267 | | 1065 | | 67 | | 247 | | 98 | | 26707 |
| 11X-407-122 | | 6 | | 1959 | | 1191 | | 53 | | 228 | | 82 | | 26938 |
| 11X-407-004 | | 24 | | 1209 | | 1188 | | 51 | | 76 | | 56 | | 27871 |
| 11X-407-044 | | 24 | | 904 | | 1012 | | 34 | | 25 | | 47 | | 28429 |
| 11X-407-059 | | 24 | | 1558 | | 1465 | | 129 | | 330 | | 79 | | 26890 |
| 11X-407-084 | | 24 | | 832 | | 980 | | 38 | | 13 | | 33 | | 28555 |
| 11X-407-089 | | 24 | | 1589 | | 1236 | | 59 | | 253 | | 61 | | 27253 |
| 11X-407-102 | | 24 | | 967 | | 944 | | 36 | | 49 | | 34 | | 28421 |
| 11X-407-122 | | 24 | | 1851 | | 1705 | | 136 | | 322 | | 100 | | 26337 |
| 11X-407-004 | | 96 | | 804 | | 1186 | | 25 | | 39 | | 41 | | 28356 |
| 11X-407-044 | | 96 | | 1480 | | 1437 | | 193 | | 105 | | 78 | | 27158 |
| 11X-407-059 | | 96 | | 750 | | 1570 | | 36 | | 91 | | 55 | | 27949 |
| 11X-407-084 | | 96 | | 630 | | 1248 | | 54 | | 20 | | 53 | | 28446 |
| 11X-407-089 | | 96 | | 1309 | | 2029 | | 140 | | 586 | | 82 | | 26305 |
| 11X-407-102 | | 96 | | 846 | | 1391 | | 64 | | 61 | | 44 | | 28045 |
| 11X-407-122 | | 96 | | 1098 | | 1532 | | 45 | | 304 | | 54 | | 27418 |
| ***Family Average by Time*** | | | | | | | | | | | | | | |
|  | **Time** | | **P1 dominant** | | **P2 dominant** | | **Overdominant** | | **Underdominant** | | **Additive** | | **Conserved** | |
|  | T0 | | 1552 | | 1125 | | 66 | | 153 | | 70 | | 27484 | |
|  | T6 | | 1498 | | 1404 | | 61 | | 153 | | 89 | | 27246 | |
|  | T24 | | 1273 | | 1219 | | 69 | | 153 | | 59 | | 27680 | |
|  | T96 | | 988 | | 1485 | | 80 | | 172 | | 58 | | 27668 | |
