# Supplementary material for: Transcriptome analysis of contrasting resistance to herbivory by Empoasca fabae in two shrub willow species and their hybrid progeny
Source: PLoS One. 2020 Jul 29;15(7):e0236586. doi: 10.1371/journal.pone.0236586 (PMC7390382; doi:10.1371/journal.pone.0236586)
Supplement: S2 Table — (DOCX) [file pone.0236586.s007.docx]

**S2 Table**. **Primer information for qPCR validation of selected genes.**

| Gene Name Abbreviation | Gene ID | Annotation | Primers  (forward/ reverse 5′-3′) | Size (bp) |
| --- | --- | --- | --- | --- |
| MUB | SapurV1A.2454s0040 | membrane-anchored ubiquitin-fold protein | TGCCAGAGGAGGAGGAGTTA | 133 |
|  |  |  | TCTTTAGGCCAGTCGGCTAC |  |
| MYB | SapurV1A.1016s0030 | MYB transcription factor in vasculature development/ xylem development | CCTGCAATTTCTTCCAGCTC | 138 |
|  |  |  | GCTTTCATGGGAGGTACCAA |  |
| CHT | SapurV1A.0194s0290 | carbohydrate transporter | GCACATGCATGACAAAAAGG | 154 |
|  |  |  | CATTAAGGGCCAATTCCTCA |  |
| Sgf11 | SapurV1A.0106s0050 | Sgf11 (transcriptional regulation protein) proteinLinks | GCGAAACCAATGGCAAGTAT | 101 |
|  |  |  | CACTATTGGTCTCCCGCAAT |  |
| ACT2 | SapurV1A.0285s0180 | actin | CAGAAAGACGCCTATGTTGG | 104 |
|  |  |  | TCCATATCATCCCAGTTGCT |  |
| NAC | SapurV1A.2717s0010 | NAC transcription factor | CCCAATGAGCAGTCCATCTT | 111 |
|  |  |  | CCAATTTGGTTTGAGGAGGA |  |
| Glu_b | SapurV1A.0427s0070 | tau class glutathione transferase GSTU6 | TGGATTGGATGGCATAGTTG | 90 |
|  |  |  | CCCAACAGTGTTACACCCATT |  |
| MYB2 | SapurV1A.0534s0230 | MYB transcription factor in plant-type secondary cell wall biogenesis | CGGAGGAAGATGACAAGCTC | 170 |
|  |  |  | TTCTTCCTGGGGTGAAAATG |  |
| FBK | SapurV1A.0571s0080 | heat shock protein-binding protein, putative | GACAGTGCAGTTTGGGACAA | 86 |
|  |  |  | CAAACAGAGCTTGGGGTCTC |  |
