# Supplementary material for: Transcriptome analysis of contrasting resistance to herbivory by Empoasca fabae in two shrub willow species and their hybrid progeny
Source: PLoS One. 2020 Jul 29;15(7):e0236586. doi: 10.1371/journal.pone.0236586 (PMC7390382; doi:10.1371/journal.pone.0236586)
Supplement: S3 Table — The table also illustrates the functional characteristics of each genes. (DOCX) [file pone.0236586.s008.docx]

**S3 Table**. **Validation of differentially expressed genes related to transcription factors (TFs), ligandins and heat shock protein of two contrasting PLH-susceptible shrub willow genotypes.** The table also illustrates the functional characteristics of each genes.

| **Gene ID** | **Gene Description** | **Time after PLH Infestation** | **RNA-Seq Normalized Expression (p < 0.05)** | | **qPCR Relative Expression** | |
| --- | --- | --- | --- | --- | --- | --- |
|  |  |  | 94006 | ‘Jorr’ | 94006 | ‘Jorr’ |
| SapurV1A.1016s0030  Abbr ID: MYB | MYB transcription factor in vasculature development/ xylem development | 0 | 1.000 | 1.916 | 1.001 | 1.300 |
|  |  | 6 hours | 1.487 | 0.726 | 1.367 | 0.787 |
|  |  | 24 hours | 3.849 | 2.157 | 3.977 | 3.937 |
|  |  | 96 hours | 3.068 | 1.391 | 3.357 | 1.459 |
| SapurV1A.0106s0050  Abbr ID: Sgf11 | Sgf11 (transcriptional regulation protein) proteinLinks | 0 | 1.000 | 0.760 | 1.007 | 0.673 |
|  |  | 6 hours | 0.957 | 0.251 | 1.304 | 0.520 |
|  |  | 24 hours | 0.861 | 0.228 | 1.180 | 0.548 |
|  |  | 96 hours | 1.070 | 0.338 | 1.036 | 0.592 |
| SapurV1A.0427s0070  Abbr ID: Glu_b | tau class glutathione transferase GSTU6 | 0 | 1.000 | 10.922 | 1.032 | 11.185 |
|  |  | 6 hours | 1.202 | 18.040 | 1.892 | 20.986 |
|  |  | 24 hours | 1.726 | 23.451 | 1.337 | 25.139 |
|  |  | 96 hours | 1.378 | 20.319 | 0.910 | 22.554 |
| SapurV1A.0534s0230  Abbr ID: MYB2 | MYB transcription factor in plant-type secondary cell wall biogenesis | 0 | 1.000 | 1.059 | 1.061 | 0.944 |
|  |  | 6 hours | 1.382 | 0.363 | 2.047 | 0.638 |
|  |  | 24 hours | 1.416 | 0.344 | 1.784 | 0.168 |
|  |  | 96 hours | 1.765 | 0.377 | 2.230 | 0.347 |
| SapurV1A.2717s0010  Abbr ID: NAC | NAC transcription factor | 0 | NA | NA | NA | NA |
|  |  | 6 hours | 1.000 | 0.102 | 1.111 | 0.174 |
|  |  | 24 hours | 1.381 | 0.064 | 1.526 | 0.145 |
|  |  | 96 hours | 1.387 | 0.063 | 1.387 | 0.160 |
| SapurV1A.0571s0080  Abbr ID: FBK | heat shock protein-binding protein, putative | 0 | NA | NA | NA | NA |
|  |  | 6 hours | NA | 98.521 | NA | 137.900 |
|  |  | 24 hours | 1.000 | 0.628 | 1.040 | 1.206 |
|  |  | 96 hours | 0.672 | 0.538 | 0.642 | 1.754 |

*r = 0.82 (P = 2.46e-11)
